# The macrophage-associated prognostic gene ANXA5 promotes immunotherapy resistance in gastric cancer through angiogenesis

**DOI:** 10.1186/s12885-024-11878-7

**Published:** 2024-01-29

**Authors:** Zhijun Hong, Peizhen Wen, Kang Wang, Xujin Wei, Wen Xie, Shihao Rao, Xin Chen, Jingjing Hou, Huiqin Zhuo

**Affiliations:** 1 Department of Gastrointestinal Surgery, School of Medicine, Zhongshan Hospital of Xiamen University, Xiamen University, Xiamen, China; 2 Xiamen Municipal Key Laboratory of Gastrointestinal Oncology, 361004 Xiamen, China; 3 The Graduate School, Fujian Medical University, 350004 Fuzhou, China; 4 Department of General Surgery, Changzheng Hospital, Navy Medical University, 415 Fengyang Road, 200003 Shanghai, China

**Keywords:** Gastric cancer, Immune infiltration, WGCNA, LASSO-Cox analysis, ANXA5

## Abstract

Gastric cancer (GC) remains a predominant form of malignant tumor globally, necessitating innovative non-surgical therapeutic approaches. This investigation aimed to delineate the expression landscape of macrophage-associated genes in GC and to evaluate their prognostic significance and influence on immunotherapeutic responsiveness. Utilizing the CellMarker2.0 database, we identified 69 immune cell markers with prognostic relevance in GC, including 12 macrophage-specific genes. A Weighted Gene Co-Expression Network Analysis (WGCNA) isolated 3,181 genes correlated with these macrophage markers. The Cancer Genome Atlas (TCGA-STAD) dataset was employed as the training set, while data from the GSE62254 served as the validation cohort. 13 genes were shortlisted through LASSO-Cox regression to formulate a prognostic model. Multivariable Cox regression substantiated that the calculated risk score serves as an imperative independent predictor of overall survival (OS). Distinct macrophage infiltration profiles, pathway associations, treatment susceptibilities, and drug sensitivities were observed between high- and low-risk groups. The preliminary validation of ANXA5 in predicting the survival rates of GC patients at 1 year, 3 years, and 5 years, as well as its expression levels were higher and role in promoting tumor angiogenesis in GC through immunohistochemistry and angiogenesis experiments. In summary, macrophage-related genes were potentially a novel crosstalk mechanism between macrophages and endothelial cells in the tumor microenvironment, and the interplay between inflammation and angiogenesis might have also offered new therapeutic targets, providing a new avenue for personalized treatment interventions.

**Supplementary Information:**

The online version contains supplementary material available at 10.1186/s12885-024-11878-7.

## Introduction

Gastric cancer (GC) is a leading global health concern, ranking fourth in incidence and fifth in mortality among all types of malignant tumors [1]. Adenocarcinoma constitutes the majority of GC cases, representing over 95% of the total [2]. Despite advances in surgical procedures, cytotoxic ·treatments, and targeted therapies, the overall prognosis for GC remains suboptimal. Emerging evidence suggests that immunotherapy holds promise for specific patient subgroups [3, 4].

Tumor microenvironment comprises a diverse array of immune cells, including macrophages, T and B lymphocytes, natural killer (NK) cells, mast cells, polymorphonuclear cells, and dendritic cells (DCs) [5]. These immune constituents play pivotal roles in tumor invasion, metastasis, and clinical outcomes [6, 7]. Specifically, tumor-associated macrophages (TAMs) serve as central innate immune components, facilitating both tumor growth and dissemination [8]. TAMs exhibit dual functions, promoting tumor growth through the M2 phenotype and suppressing it via the M1 phenotype [9]. Despite extensive research on TAMs in GC, studies related to the genes associated with TAMs in establishing prognostic models for GC remain limited. Genes associated with TAMs reshape the tumor microenvironment by modulating inflammatory responses [10], potentially leading to increased drug resistance and promoting various cancer cell growth phenotypes.

The Annexin A (ANXA) family is a subset of the phospholipid-binding protein superfamily, categorized into five groups: A, B, C, D, and E [11]. Comprising 12 members in humans, the ANXA family is involved in diverse cellular functions that intersect with fundamental tumor characteristics, such as cell proliferation, apoptosis, vesicular transport, and signal transduction [12, 13]. Emerging research has demonstrated that dysregulated expression of ANXA family members is intricately linked to tumorigenesis and tumor progression [14–16]. Although prior studies have indicated a regulatory interplay between ANXA5 and immune cells [17, 18], its specific molecular mechanisms in the context of GC remain largely unexplored.

In this study, we conducted a comprehensive analysis of immune cell signature genes from publicly available databases. A LASSO risk model was constructed based on macrophage signature genes, and its predictive efficacy was validated. Furthermore, we analyzed the divergence in immune infiltration between high- and low-risk groups as defined by the prognostic model. Focusing on the gene ANXA5, we experimentally verified its elevated expression levels in GC and its contributory role in carcinogenesis.

## Materials and methods

### Access to immune cell signature genes

Specific cell type marker genes were retrieved from the CellMarker2.0 database [19]. The cancer types specified for filtering were “GC” and “Gastric Adenocarcinoma.” A preliminary screening yielded 551 immune cell markers, which were further refined to 69 immune cell signature genes via univariate Cox analysis. The Sankey Plot was employed to elucidate the associations between immune cells and characteristic genes.

### Description of data resources

Sequencing data for mRNA and clinical information were sourced from The Cancer Genome Atlas (TCGA) and GTEx databases. Duplicate entries across all datasets were removed, followed by Log2(TPM + 1) transformation to ensure data consistency and availability. A validation cohort, complete with expression profiles and prognostic data, was acquired from the GEO database.

### Weighted gene co-expression network analysis (WGCNA)

We preprocessed the raw gene expression data to eliminate low-quality samples and genes. Specifically, we calculated the Median Absolute Deviation (MAD) for each gene across all samples. The MAD values were utilized to estimate the variability in gene expression across different samples. Genes with MAD values below the 50th percentile were excluded to minimize noise and enhance the accuracy of subsequent analyses. Following gene filtering, we employed the “goodSamplesGenes” of WGCNA package to further eliminate samples that may contain outliers or potentially compromise the integrity of the co-expression network. This method primarily relies on the coherence of samples and genes to identify and eliminate outliers. Next, we generated a scale-free co-expression network with WGCNA package. To ensure the scale-free nature of the network, we selected an appropriate soft-thresholding power,β, through scale-free topology model fitting. This threshold was applied to transform the Pearson correlation coefficient matrix between genes into a weighted adjacency matrix, specifically A_mn_=|C_mn_|^β^, where A_mn_ represents the adjacency between gene m and gene n, and C_mn_ is their Pearson correlation. Furthermore, we calculated the Topological Overlap Matrix (TOM) to assess the connectivity of each gene with all other genes in the network. The TOM was utilized to strengthen the adjacency matrix, enabling more accurate module identification. Using average linkage hierarchical clustering, we identified a set of gene modules and computed the module eigengene for each module. In this stage, it is customary to compute the initial principal component of the expression patterns of all genes within the module. Lastly, we employed Spearman correlation to estimate the associations between the module eigengenes and macrophage traits. Based on the correlations and biological relevance of the module traits, certain highly similar modules were merged using a module tree cutting line.

### Construction and validation of a macrophage feature gene-related prognostic signature

Genes implicated in Overall Survival (OS) were initially selected from a pool of 24 hub genes, identified based on their high connectivity within the co-expression network modules. To minimize redundancy and prevent overfitting, a LASSO Cox regression analysis was subsequently conducted on these selected genes using the TCGA training set via the ‘glmnet’ R package. From this, a risk score model was constructed using coefficients estimated for 13 selected genes. Patients from the TCGA dataset were stratified into high-risk and low-risk categories based on the calculated risk scores. Kaplan-Meier (K-M) survival curves were generated to visualize the prognostic differences between these two risk groups. To validate the independent predictive capability of this macrophage feature gene-based signature, both univariate and multivariate Cox regression analyses were performed. These analyses culminated in the construction of a nomogram that incorporated both the risk scores and additional clinical variables, such as age and tumor stage. The model’s predictive accuracy for OS was further evaluated using a calibration plot, which demonstrated a high degree of concordance between the nomogram-predicted and actual observed OS.

### Signaling pathway analysis

Gene sets representing 105 key signaling pathways were curated from the Molecular Signatures Database (MsigDB). These pathways were strategically selected for their relevance to critical biological processes such as Angiogenesis, Collagen Formation, and Extracellular Matrix (ECM) interactions, among others. Enrichment scores for each selected pathway were computed using the Single Sample Gene Set Enrichment Analysis (ssGSEA) algorithm, available in the ‘GSEA’ package for R. This particular method was employed for its robustness in generating individualized pathway activity profiles on a per-sample basis. To substantiate the relationship between the risk scores—originally derived from our LASSO Cox regression model—and the enrichment scores of these pathways, we performed Spearman’s rank correlation analysis. The correlation metrics were subsequently visualized using specialized graphical representations, generated through the ‘ggplot2’ package in R.

### Immune infiltration, immunotherapy response, and drug sensitivity assessment

We employed a comprehensive suite of 12 immune infiltration algorithms, utilizing R packages such as ‘GSVA’ for ssGSEA and ‘immunedeconv’ for algorithms including TIMER [20], xCell [21], MCP-counter [22], CIBERSORT [23], EPIC [24], and quanTIseq [25]. To calculate immune cell enrichment scores, the ssGSEA algorithm was applied using immune cell markers from three different sets: 24 markers provided by Bindea G et al. (2013) [26], 13 markers from Safonov A et al. (2017) [27], and 28 markers by Charoentong P et al. (2017) [28]. We also employed the TIP (Tracking Tumor Immunophenotype) algorithm to analyze the seven steps of the cancer-immunity cycle and to infer the proportions of various tumor-infiltrating immune cells [29]. ImmuneCellAI was used to estimate the proportions of 18 types of T cells and six other categories of immune cells, including B cells, NK cells, monocytes, macrophages, neutrophils, and dendritic cells (DCs). This algorithm also predicts patient responses to immune checkpoint inhibitor therapy [30]. The ESTIMATE algorithm was implemented to infer tumor purity, as well as stromal and immune cell admixture within the tumor microenvironment [31]. Finally, the Mann-Whitney U test (also known as the Wilcoxon rank-sum test) was employed to ascertain the statistical significance of differences in immune cell infiltration between the two subgroups. Significance levels were denoted as follows: ns for *p* ≥ 0.05; * for *p* < 0.05; ** for *p* < 0.01; *** for *p* < 0.001.

The TIDE algorithm was utilized for predicting potential immunotherapy responses. Data pertaining to IC50s of 481 small-molecule probes and drugs, along with corresponding gene expression profiles, were obtained from the Cancer Therapeutics Response Portal (CTRP) [32]. Pearson correlation analysis was used to establish the relationship between gene expression and drug IC50 values, and the results were displayed using bubble plots.

### Analysis of genomic heterogeneity and gene expression relationship

Through the use of the “Maftools” R package, we presented distinct mutational landscapes in high-risk and low-risk groups within STAD by analyzing the STAD somatic mutation data downloaded from TCGA. Tumor purity data for each sample were obtained from a previous study [33]. Likewise, data on homologous recombination deficiency (HRD) and loss of heterozygosity (LOH) for each tumor were also sourced from the same previous study [33]. The Mann-Whitney U test, also known as the Wilcoxon rank-sum test, was employed to ascertain differences in genomic heterogeneity indicators among different samples.

### Survival and gene expression analysis

Given the continuous nature of gene expression data, we utilized the surv_cutpoint function from the survminer R package to define an optimal cut-off value. This enabled us to stratify the gene expression levels into ‘high’ and ‘low’ categories for subsequent analyses. Kaplan-Meier survival curves were generated using the survfit function from the survival package in R. These curves depicted Overall Survival (OS), as well as cumulative event and hazard rates, offering a comprehensive view of patient outcomes over time. The visualization was enhanced using the ggsurvplot function, allowing for more intuitive interpretations.

To quantify the relative risks associated with the high and low gene expression groups, Hazard Ratios (HR) were computed and visualized using error line plots, providing a straightforward representation of the associated uncertainties. Additionally, the Kruskal-Wallis test was employed to assess the statistical significance of differential gene expression between normal and tumor tissues, leveraging data from both the TCGA and GTEx databases.

### Tissue microarray and immunohistochemistry

Following deparaffinization steps, including baking, xylene treatment, and a gradient alcohol series, antigen retrieval was conducted on tissue chip specimens (HStmA160CS01, OUTDO Biotech, Shanghai, China) using citrate buffer. Subsequently, the samples underwent the following procedures: removal of hydrogen peroxidase with a 3% hydrogen peroxide solution for 10 min, followed by blocking with goat serum for 10 min. The ANXA5 primary antibody (Proteintech, Wuhan, China) was then incubated overnight at 4 °C, and immunohistochemistry staining was carried out utilizing an immunohistochemistry kit from Maixin Biotech (Fuzhou, China).

The staining degree was assessed by categorizing the percentage of stained area into four grades: Grade 1 (≤ 25%), Grade 2 (25–50%), Grade 3 (50–75%), and Grade 4 (≥ 75%). Simultaneously, staining intensity was classified into four grades: Grade 0 (no staining), Grade 1 (weak positivity), Grade 2 (moderate positivity), and Grade 3 (strong positivity). These two scores were then multiplied together to generate a weighted score, which ranged from 0 to 12. The results were independently evaluated by two pathologists based on both staining intensity and degree.

### Scratch, migration, and proliferation experiments

After digestion of SGC-7091 (Chinese Academy of Science, Shanghai, China), they were seeded into a 6-well plate. When the cell density reached confluence, a scratch was made using a 10 µl pipette tip. Subsequently, the medium was changed, and photographs were taken at 24 h, 48 h, and 72 h to record the progress. For the Transwell invasion assay, 700 µl of complete culture medium was placed in the lower chamber of a 24-well plate. Then, the chamber was placed in the incubator, and the cell suspension with a density of 1 × 10^5 cells per 200 µl was added to the upper chamber. After 16 h of incubation, cells were fixed with paraformaldehyde and stained with crystal violet. For the EdU proliferation assay, you can refer to the specific steps outlined in the BeyoClick™ EdU Cell Proliferation Kit.

### Angiogenesis detection

Matrigel was carefully thawed overnight at 4 °C to ensure uniformity. Prior to use, pipette tips and culture plates were preconditioned by chilling them at -20 °C. A precisely measured volume of 150–200 µL of Matrigel (Corning,Lowell, MA, USA) was then dispensed into each well of a 48-well culture plate. To avoid potential artifacts, the absence of air bubbles was meticulously confirmed before proceeding. The plates were subsequently incubated at 37 °C for a minimum of 2 h to facilitate complete Matrigel solidification. Endothelial cells were harvested using enzymatic digestion and their density meticulously adjusted to 3 × 10^5 cells/mL. A 200 µL aliquot, containing approximately 2 × 10^4 cells, was seeded into each pre-coated well. Angiogenic activity was then assessed following an incubation period ranging from 8 to 16 h.

### Cell culture and transfection

EA.hy 926 (Human umbilical vein cells fused cells) (Chinese Academy of Science, Shanghai, China) were cultured in DMEM/F12 (Biological Industries) supplemented with 10% fetal bovine serum(Sigma, St. Louis, MO, USA) and 100 U/ml penicillin and 100 µg/ml streptomycin(Biological Industries, Kibbutz Beit Haemek, Israel) at 37 °C with 5% CO2. In this study, we employed Turbofect (Thermo Scientific, Waltham, MA, USA) to successfully introduce ANXA5 siRNA and control sequences into the EA.hy926. The following sequences were employed:

ANXA5-siNC:5’-UUCUCCGAACGUGUCACGUTT-3’(sense),

5’-ACGUGACACGUUCGGAGAATT-3’(antisense).

ANXA5-si#1: 5’-GGAGCUGGAACAAAUGAAATT-3’(sense),

5’-UUUCAUUUGUUCCAGCUCCTT-3’(antisense).

ANXA5-si#2: 5’- GACCUGAAAUCAGAACUAATT-3’(sense),

5’- UUAGUUCUGAUUUCAGGUCTT-3’(antisense).

### Western blot assay

Cells were lysed using RIPA buffer (Solarbio, Beijing, China) with a 1:100 dilution of Cocktail (Lablead, Beijing, China). Protein concentration was determined via the BCA method and samples were prepared for SDS-PAGE using 4×LDS sample buffer (Genscript, Nanjing, China). Following electrophoresis, proteins were transferred to PVDF membranes, which were then blocked and incubated with primary ANXA5 antibodies with a 1:100 dilution (Cat No: 11060-1-AP, Proteintech, Wuhan, China). HRP-conjugated secondary antibodies were applied, and after the incubation with primary antibodies, the membranes were washed with TBST for 30 min and then incubated with HRP-conjugated secondary antibodies at a 1:10,000 dilution for one hour at room temperature. Visualization was achieved using ECL kits following a subsequent 30-minute wash with TBST.

### Statistical analysis

The entire dataset was analyzed using R software (version 4.1.0). Pearson correlation was utilized to identify significant correlations between variables. Kaplan-Meier survival curves were compared using the log-rank test. The “timeROC[0.4], ggplot2[3.3.6]” R package facilitated ROC analyses. The significance levels were defined as follows: *P* > = 0.05; *P < 0.05; **P < 0.01; ***P < 0.001; ****P < 0.0001.

## Result

### Identification of key macrophage-related genes

**Fig. 1:**
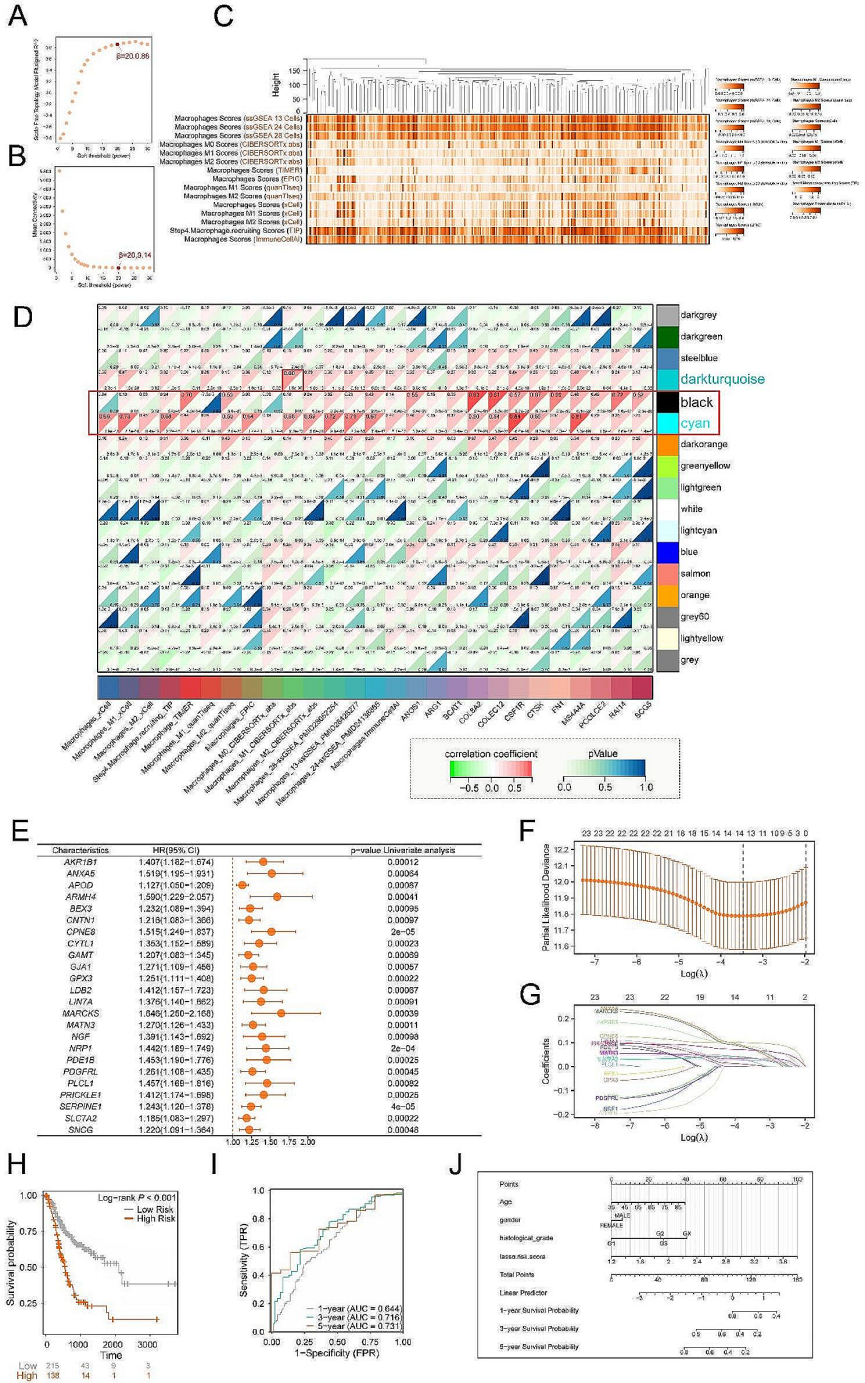
Constructing WGCNA and prognosis model with LASSO regression. (**A**) The relationship between the scale-free fit index (y-axis) and the soft thresholding power (x-axis). (**B**) The relationship between average connectivity (degree, y-axis) and soft thresholding power. (**C**) Sample clustering and immune infiltration characteristics. (**D**) To generate a correlation heatmap illustrating the correlation between module genes and macrophage infiltration as well as marker genes. (**E**) Selecting genes associated with gastric cancer prognosis with single factor regression analysis from key module genes identified by WGCNA. (**F-G**) LASSO Cox regression to identify the top 13 genes associated with prognosis. (**H**) Comparison of median survival time between high-risk and low-risk groups using KM survival curves. (**I**) Time-dependent receiver operating characteristic (ROC) analysis for evaluating the performance of the prognostic model. (**J**) Column chart depicting prognosis prediction for gastric cancer patients based on Cox regression analysis

Based on CellMarker2.0 database, we obtained 551 corresponding marker genes of immune cells. Subsequently, using univariate Cox regression analysis, we identified 69 marker genes significantly associated with OS of gastric cancer (GC) patients from the TCGA. We visualized the relationship between these prognostic genes and cell types using a Sankey plot (Figure S1). To investigate the correlation between macrophage and GC prognosis, we selected prognostic marker genes of macrophage. After obtaining the transcriptomic dataset of GC, we checked for missing values and removed outlier samples. Subsequently, sample clustering was performed, the infiltration score of macrophages was evaluated (Fig. 1C). Based on the criterion of approximate scale-free topology, we selected 20 as the appropriate soft threshold by network topology analysis (Fig. 1A-B). Using this threshold, we constructed a weighted scale-free co-expression network. The network construction and module identification were performed hierarchical cluster analysis. The results were represented by branches of a clustering tree, which generated modules of different colors (Figure S2A). These modules were obtained based on the criteria set for cutting the clustering results. We merged modules with a distance smaller than 0.25 and set the minimum module size to 30. In the end, we obtained a total of 17 co-expression modules.

Additionally, we generated a module correlation heatmap (Figure S2B) using the GC transcriptomic data to depict the magnitudes of correlations between different co-expression modules. The color intensity in the heatmap represents the correlation between modules. To link modules with phenotypes, we initially presented macrophage scores from various algorithm sources (Figure S2C). It is evident that the high-risk group exhibits consistently higher macrophage scores. We calculated the relationship between all modules and clinical features to determine the modules significantly associated with macrophage characteristics. This analysis involved calculating the Spearman correlation between module eigengenes and macrophage characteristics (Fig. 1D). Finally, we identified three modules, black, cyan, and darkturquoise, that were significantly positively correlated with macrophage features. Genes within these three modules may play a key role in immune infiltration of macrophages.

### Constructing a clinical predictive model with lasso regression based on macrophage related genes

We extracted 3181 genes from three modules and performed univariate Cox regression analysis to identify genes significantly associated with OS of GC (*p* < 0.001) (Fig. 1E). Lasso Cox regression analysis was performed on the 24 identified prognostic module genes in TCGA training set (Fig. 1F-G). Finally, we identified 13 OS-associated genes along with their corresponding coefficients (Figure S3A). The risk score was calculated as follows: (0.09915 × Exp of CPNE8) + (0.08184 × Exp of AKR1B1) +(0.07190 × Exp of MARCKS) + (0.07070 × Exp of ANXA5) + (0.06839× Exp of SERPINE1) + (0.03589× Exp of GAMT) + (0.03384 ×Exp of SNCG) + (0.03292 × Exp of MATN3) + (0.03260 × Exp of SLC7A2) + (0.02664 × Exp of CYTL1) + (0.01933 × Exp of LIN7A) + (0.00676 × Exp of GJA1) + (0.00664 × Exp of APOD), where Exp represents mRNA expression.

We computed the risk score for each sample and subsequently stratified all samples into high-risk and low-risk categories. Subsequently, we conducted Kaplan-Meier survival analysis to examine the survival disparities between these high-risk and low-risk groups within the TCGA-STAD training dataset. Our findings indicated that the high-risk group exhibits a less favorable prognosis (Fig. 1H). We conducted time-dependent ROC analysis to assess the predictive accuracy of the model. In training set, the AUC values for 1-, 3-, and 5-year OS were 0.644, 0.716, and 0.731, respectively (Fig. 1I). The distribution of risk scores, risk groups, survival outcomes, and molecular expression in training set for the prognostic model is displayed (Figure S3B). Based on Cox regression, we employ multivariate regression to construct a nomogram for predicting 1–5 years OS (Fig. 1J). By calculating the scores for variables, we can make predictions on the prognosis of patients. According to the multivariable Cox model, we have obtained the coefficients for each variable by integrating clinical variables and risk scores from 13 OS-associated genes (Figure S3C).

Utilizing this model, we computed fresh risk scores for each sample and subsequently carried out Kaplan-Meier survival analysis as well as ROC analysis. The outcomes consistently demonstrate that the high-risk group exhibits a less favorable prognosis (Figure S3E). The corresponding AUC values for predicting the prognosis at 1, 3, and 5 years are 0.667, 0.750, and 0.745, respectively (Figure S3D). We conducted univariate and multivariate Cox regression analyses on TCGA data to explore the association between clinical characteristics and risk scores (Figure S3F-G). Based on the above, the incorporation of clinical variables along with a 13-gene risk score into the multivariate Cox model showcases a strong predictive capacity.

Following this, we applied a Lasso-risk score to our validation set (GSE62254_ACRG), utilizing a comprehensive multivariate Cox regression framework that amalgamated the Lasso-derived risk score with relevant clinical parameters. The ensuing outcomes were graphically represented through a forest plot and complemented by a calibration curve, serving to substantiate the reliability and robustness of our predictive model (Figures S4A-B). A subsequent re-evaluation of each clinical variable led to the assignment of revised coefficients, which are illustrated in Figure S4C. To facilitate a more nuanced understanding, both univariate (Figure S4D) and multivariate (Figure S4E) Cox regression analyses relative to these clinical variables were elucidated via corresponding forest plots. Capitalizing on these newly derived coefficients, we proceeded to recalibrate individual risk scores within the validation cohort. The refined model displayed noteworthy predictive potency, as corroborated by its performance indicators for forecasting 1-, 3-, and 5-year overall survival (OS) rates, which stood at 0.884, 0.801, and 0.774, respectively (Figure S4F). Congruent with these metrics, the subset of the cohort earmarked as high-risk exhibited a markedly adverse prognostic outcome (Figure S4G). In an endeavor to rigorously assess the prognostic fidelity of our constructed model, particularly in juxtaposition with clinical variables, we undertook Decision Curve Analysis (DCA) for the 1-, 3-, and 5-year survival estimations. The composite model, which synergized “Clinical Variables + RiskScore,” outperformed alternative models in prognostic precision, as evidenced in Figures S4H-J.

### Evaluating immune infiltration patterns and mutation analysis in high- and low-risk groups

To evaluate the macrophage infiltration between the high-risk group and the low-risk group, 12 algorithms were employed to assess immune infiltration pattern. The algorithms employed include xCell, CIBERSORT, ssGSEA (3 gene sets), ImmuneCellAI, ESTIMATE, TIP, MCP-counter, quanTIseq, TIMER, and EPIC. Using all of the algorithms mentioned, significant differences has been found in the immune infiltration patterns between the high-risk group and the low-risk group (Figure S5A-L). Specifically, when analyzing the immune infiltration cell types, the algorithms ssGSEA, CIBERSORT, ImmuneCellAI, xCell, quanTIseq, TIMER, EPIC and TIP have revealed significant variations in macrophage infiltration when comparing the high- and low-risk groups. It was quite apparent that across all algorithms, the high-risk group exhibited higher macrophage infiltration scores. Furthermore, in the ImmuneCellAI, 24-ssGSEA, 28-ssGSEA, MCP-counter, and xCell algorithms, it could be observed that various immune cell types such as mast cells, T cells, and others had higher infiltration scores in the high-risk group compared to the low-risk group. This observation is consistent with the previous analysis results, which indicates a significant association between the 13 OS-associated genes and the functional role of macrophages in GC.

High TMB (Tumor Mutational Burden) is associated with immune therapy response and sustained clinical benefits. Therefore, we investigated the discriminatory ability of the macrophage signature genes in the somatic mutation data of TCGA-STAD cohort. Firstly, we identified the genes with the most differential mutations among different risk groups, including TTN, TP53, MUC16, LPR1B, etc. (Figure S6A). Subsequently, we further investigated the single nucleotide variants (SNV) and mutation frequencies of these feature genes. LIN7A, GJA1, and SLC7A2 are the three genes with the most frequent SNV mutations and overall gene mutations in the STAD data (Figure S6B-C). Finally, our analysis revealed that the high-risk group exhibited lower tumor purity, along with a higher frequency of homologous recombination deficiency (HRD) and loss of heterozygosity (LOH) events (Figure S6D-F). More diverse immune microenvironment and lower tumor purity were associated with increased mutation rates [34]. Higher HRD was linked to germline mutations in GC [35], and higher LOH events served as predictive markers for neoadjuvant therapy [36].

### Comprehensive analysis of risk stratification, pathway enrichment, immune signatures, and drug sensitivity in gastric cancer

**Fig. 2:**
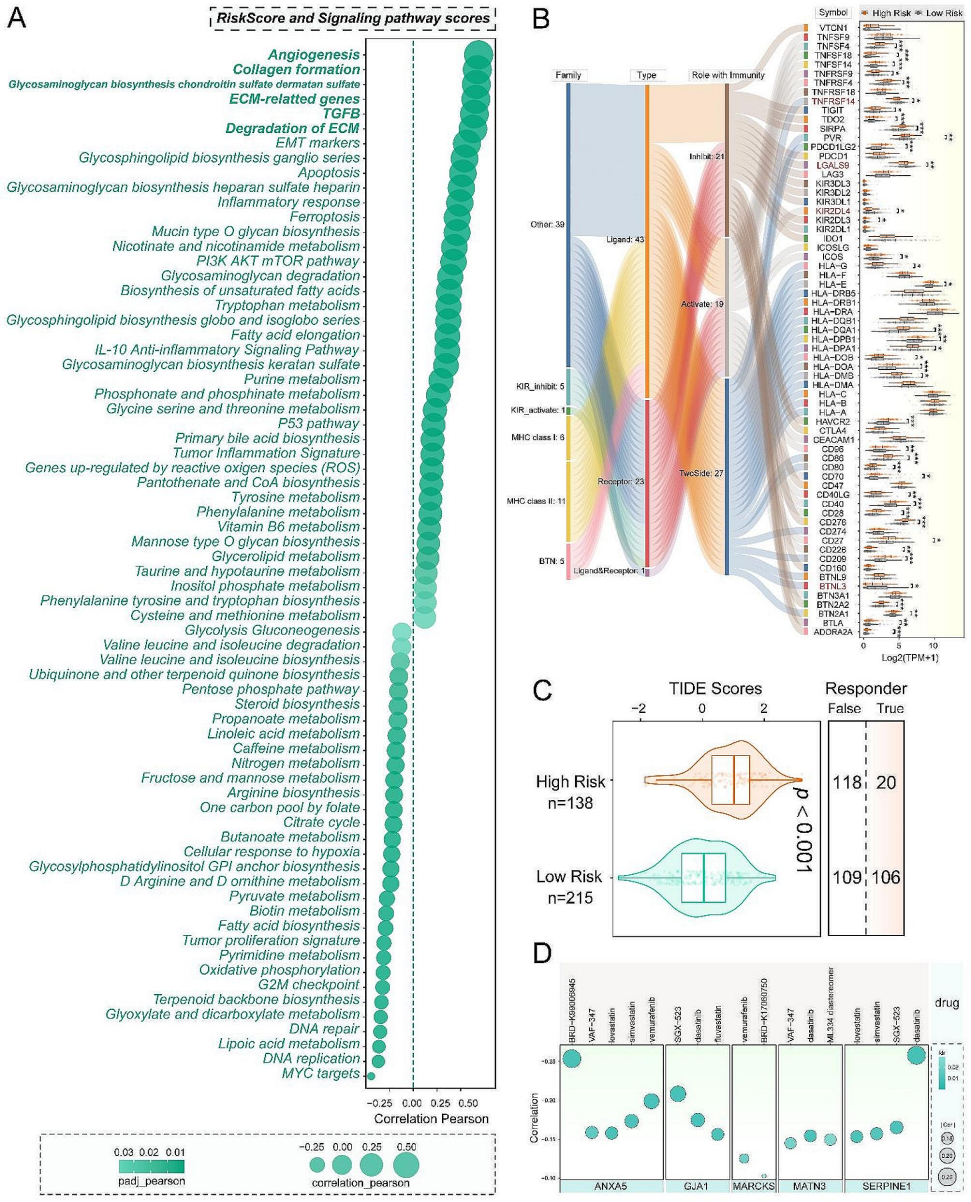
Comprehensive analysis of risk stratification, pathway enrichment, immune signatures, and drug sensitivity in gastric cancer. (**A**) risk score assessment of tumor-related pathway scores. (**B**) Correlation between different risk groups and the expression of immune-related genes. (**C**) Tumor immune dysfunction and exclusion (TIDE) scores in different risk groups. (**D**) prediction of drug sensitivity of prognostic-related genes

Performing analysis with the R package “GSVA” on a set of 105 common tumor pathway gene sets with the method parameter set to “ssgsea”. We calculated the enrichment scores for each sample across the 105 common tumor pathway gene sets and then assessed the correlation between the risk score and pathway enrichment scores using Pearson correlation (Fig. 2A). It can be observed that there is a significant correlation between the high-risk group and pathways such as Angiogenesis, Collagen formation, and ECM-related genes. High expression of immune checkpoint genes generally correlates with a better response to immunotherapy. Therefore, we analyzed the expression of immune checkpoint genes between the high-risk and low-risk groups (Fig. 2B). As seen in the figure, immune checkpoint genes marked in red showed higher expression in the low-risk group, suggesting that these genes might be potential targets for immunotherapy in the low-risk group. We conducted Tumor Immune Dysfunction and Exclusion (TIDE) analysis and found that the low-risk group had lower scores (Fig. 2C). The TIDE score reflects sensitivity to immune checkpoint inhibitors, indicating that the low-risk group is more likely to benefit from immune-based therapeutic approaches. We investigated the relationship between mRNA expression of the core genes in prognostic model and the sensitivity of drugs measured by IC50 values. The results revealed that ANXA5, GJA1, MARCKS, MATN3, and SERPINE1 exhibit sensitivity to multiple drugs (Fig. 2D). Specifically, ANXA5 shows sensitivity to drugs such as BRD-K99006945, VAF-347, lovastatin, simvastatin, and vemurafenib.

### ANXA5 as a prognostic marker and therapeutic target in gastric cancer based on insights from comprehensive analysis

Further analysis revealed that each of the 13 prognostic model genes individually correlates with the prognosis of GC, OS was selected as the prognosis type. Moreover, high expression of each gene is positively associated with poor prognosis (Figure S7A-M). We visualized the Hazard Ratio (HR) values (Figure S7N). We conducted expression analysis of these 13 genes based on TCGA and TCGA-GTEx data. It is evident that most of them are highly expressed in cancer, with ANXA5, SERPINE1, MARCKS, and MATN3 being particularly significant in their expression levels (Figure S7O). Based on risk factors, drug sensitivity screening, and the degree of differential expression between cancer and normal tissues, we have identified ANXA5, MARCKS, and SERPINE1 as candidates warranting further research. MARCKS has been found to exhibit significant pro-cancer effects in various cancers [37, 38]. Similarly, SERPINE1 has been extensively studied in the context of tumors and has been confirmed to play a substantial role in promoting angiogenesis [39, 40]. However, the role of ANXA5 in the development of GC is unclear. Therefore, we have chosen ANXA5 as the focus of our next research endeavor.

**Fig. 3:**
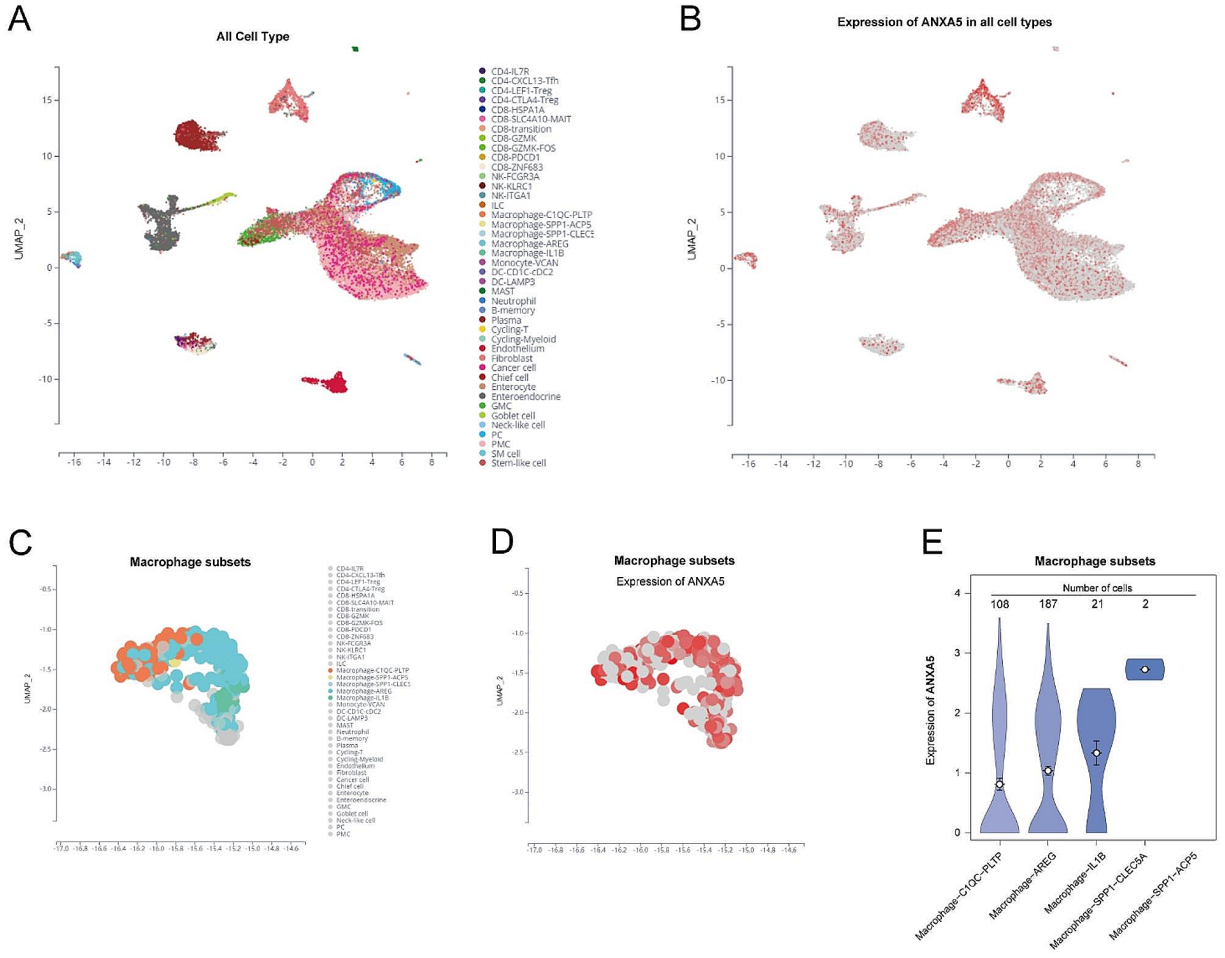
The expression landscape of ANXA5 at the single-cell level. (**A**) The gastric cancer single-cell dataset GSE134520 underwent dimension reduction and clustering using the scTIME portal. (**B**) Expression levels of ANXA5 across different cell types. (**C**) Subclassification of macrophage subtypes. (**D-E**) Expression levels of ANXA5 in macrophage subtypes

In the Shiny framework of the interactive website, we employed the scTIME portal developed by Hong F and colleagues [41] to analyze the single-cell dataset GSE134520 sourced from GEO. We annotated various cell types, ultimately generating a single-cell landscape of GC (Fig. 3A). We also analyzed the expression levels of ANXA5 in different cell types within the microenvironment (Fig. 3B). It could be observed that ANXA5 is widely expressed, with higher abundance in fibroblasts and macrophages. Furthermore, we subdivided macrophages into various subgroups (Fig. 3C) and analyzed the expression of ANXA5 within these subgroups (Fig. 3D). Notably, among macrophages, the subgroups Macrophage − C1QC − PLTP, Macrophage − AREG, Macrophage − IL1B, and Macrophage − SPP1 − CLEC5A exhibited the highest levels of ANXA5 expression (Fig. 3E).

**Fig. 4:**
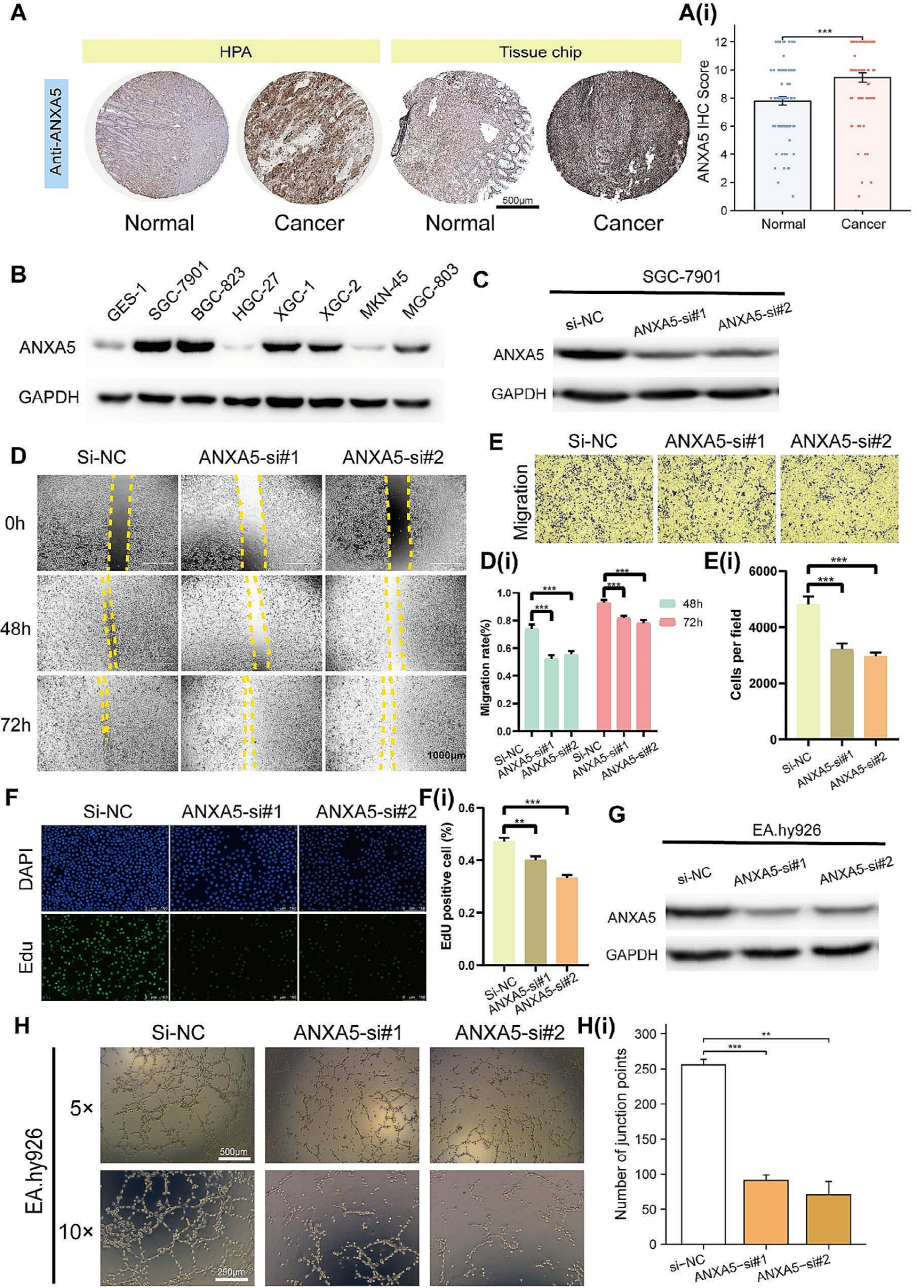
High expression of ANXA5 promotes the progression of gastric cancer. (**A**) Immunohistochemistry results of ANXA5 in the Human Protein Atlas (HPA) database and tissue chips. (**B**) Expression levels of ANXA5 in gastric cancer cell lines and normal gastric mucosal epithelium (GES-1). (**C**) Silencing ANXA5 with small interfering RNA (siRNA) in SGC-7901. Scratch assay (**D**) and migration assay (**E**) as well as proliferation assay (**F**) results of SGC-7901 cells after ANXA5 silencing. (**G**) Silencing ANXA5 with small siRNA in EA.hy926 cells. (**H**) ANXA5 silencing inhibits angiogenesis. i: Quantitative statistical results of the corresponding experimental outcomes. **p < 0.01; ***p < 0.001. Full-length gels are presented in Supplementary Fig. 5

In the Human Protein Atlas (HPA) database, ANXA5 was found to be expressed at a higher level in GC compared to adjacent tissue, and this result was validated with tissue microarray analysis (Fig. 4A). The tissue microarray comprises 80 cancer specimens and 80 paired adjacent specimens. The immunohistochemistry score for ANXA5 in GC was higher than that in the adjacent non-cancerous tissue. In GC cell lines, including SGC-7901, BGC-823, XGC-1, XGC-2 and MGC-803, the expression levels of ANXA5 were higher than those in GES-1 (Human Gastric Epithelial Cells) (Fig. 4B). The HGC-27 and MKN-45 cell lines, which exhibited lower differentiation, had lower ANXA5 expression. This suggests a correlation between ANXA5 expression and the degree of differentiation of gastric cancer cells. Subsequently, in order to explore the potential functions of ANXA5, we conducted ANXA5 siRNA interference in SGC-7901 (Fig. 4C) and EA.hy 926 (Human umbilical vein cells fused cells) (Fig. 4G). The scratch assay and transwell migration assay were performed to evaluate silencing of ANXA5 impact on the migration ability of SGC-7901. It can be observed that silencing of ANXA5 significantly inhibited the migration ability of SGC-7901 (Fig. 4D-E). Reduced expression of ANXA5 also inhibits the proliferation ability of SGC-7901 (Fig. 4F). After the suppression of ANXA5 expression, the EA.hy 926’s ability of angiogenesis was significantly inhibited (Fig. 4H). Anti-angiogenic therapy presents an exciting approach to cancer treatment, particularly when combined with strategies to overcome immune tolerance. The combination of intratumoral vasculature and inflammation could be used as predictors of immunotherapy [42].

**Fig. 5:**
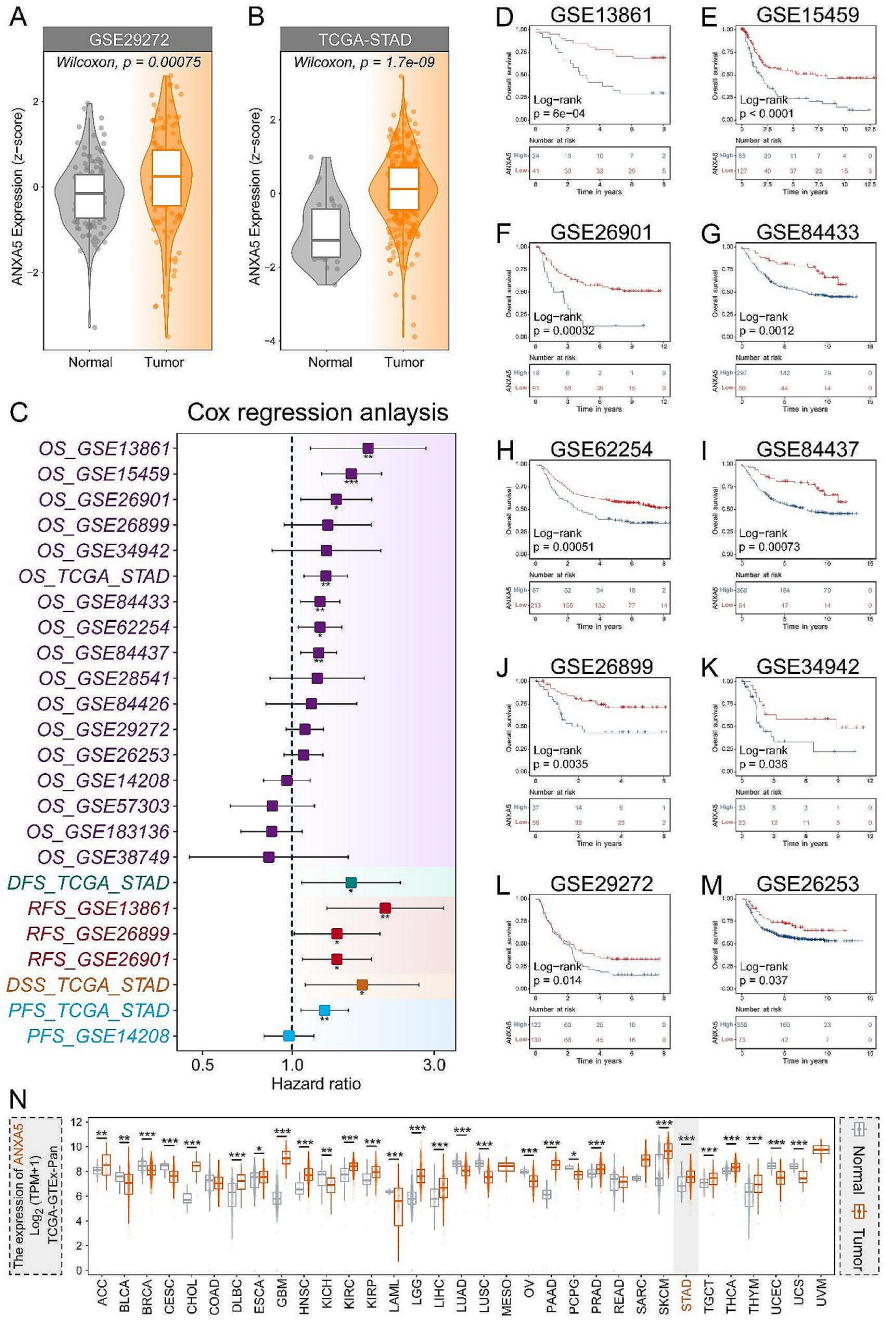
Expression Analysis of ANXA5. (**A-B**) Expression profiles of ANXA5 in GSE29272 (**A**) and TCGA-STAD (**B**). (**C**) Cox regression analysis across multiple datasets to assess the impact of ANXA5 on the prognosis of gastric cancer patients; (**D-M**) Evaluation of the effect of ANXA5 expression on the OS of gastric cancer patients in 10 GEO datasets with Kaplan-Meier (KM) curves; (**N**) Pan-cancer analysis of ANXA5 expression in TCGA

**Fig. 6:**
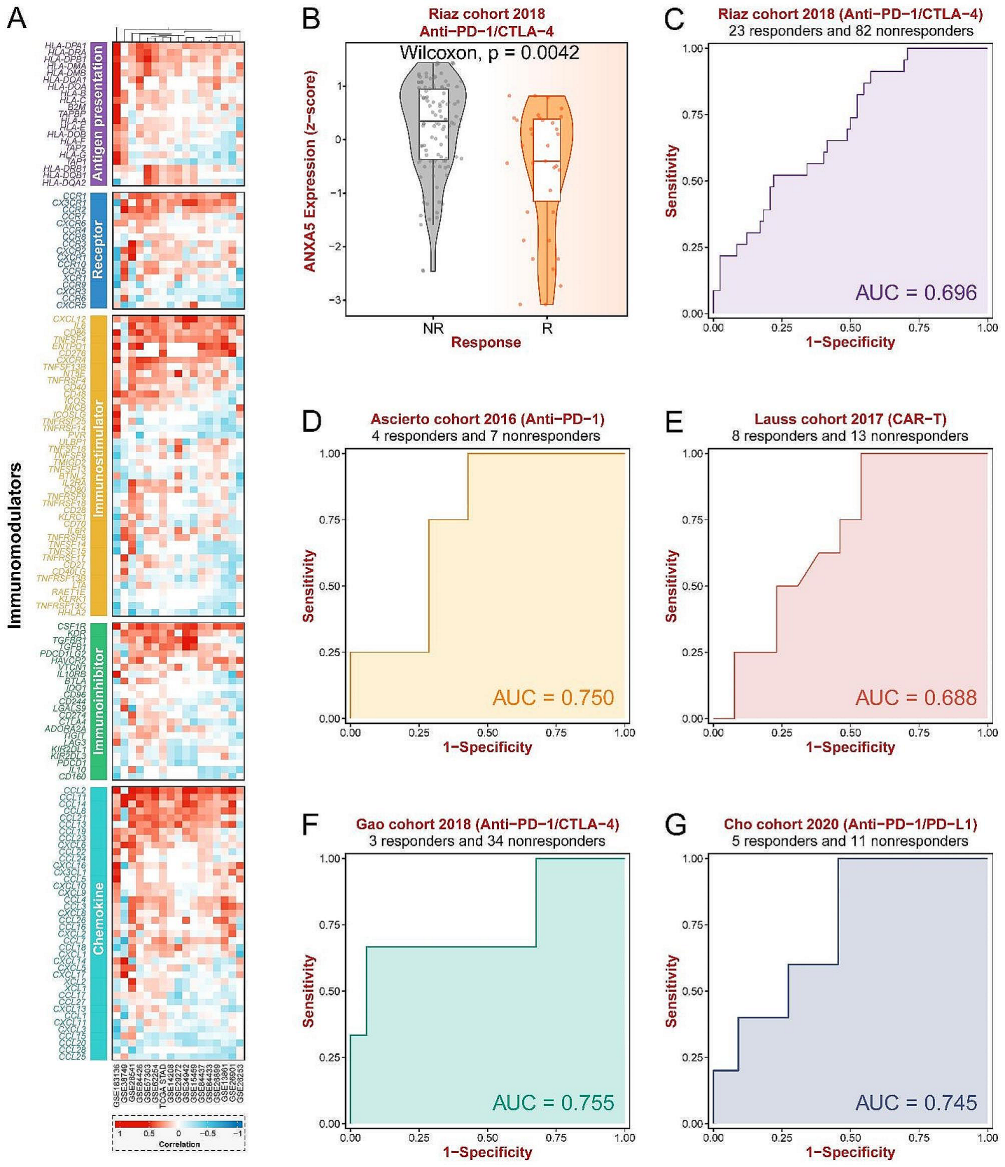
Predicting the Impact of ANXA5 Expression on Immunotherapy Response in Gastric Cancer Patients. (**A**) Analyzing the correlation between ANXA5 and the expression of immune-related genes in 17 public datasets; (**B**) Assessing the correlation between ANXA5 expression and the response to anti-PD-1/CTLA-4 therapy in the Riaz cohort 2018; (**C-G**) Evaluating the diagnostic performance of ANXA5 expression in predicting immunotherapy response in five cohorts

In both the TCGA-STAD and GSE29272 datasets, ANXA5 was found to be highly expressed in GC (Fig. 5A-B). Subsequently, through the analysis of 10 GC datasets from GEO, we discovered a significant correlation between high expression of ANXA5 and adverse prognosis in GC, including OS, disease-free survival (DFS), recurrence-free survival (RFS), disease-specific survival (DSS), and progression-free survival (PFS) (Fig. 5C-M). Pan-cancer analysis revealed widespread high expression of ANXA5 in various types of tumors (Fig. 5N). To assess the correlation between ANXA5 and immunotherapy in the GC dataset mentioned above, we evaluated the expression correlation of ANXA5 with five types of immunomodulators in STAD cohorts (Fig. 6A). These immunomodulators include antigen presentation, receptors, immune stimulators, immune inhibitors, and chemokines. Such correlations suggest that ANXA5 may regulate the tumor microenvironment by influencing these immunomodulators. Conversely, we also noted some significant negative correlations, which may indicate that ANXA5 plays a role in inhibiting the function of immunomodulators in certain circumstances. We found that ANXA5 expression was higher in the non-responder group in anti-PD-1/CTLA − 4 therapy of Riaz cohort 2018 (Fig. 6B). The ROC curve was then plotted with ANXA5 expression value as the independent variable and clinical information (immunotherapy response or not) as the dependent variable. AXA5 was found to have diagnostic efficacy in predicting immunotherapy response in multiple cohorts (Fig. 6C-G).

## Discussion

Macrophages, as participants in innate immune responses, constitute a major component of leukocyte infiltration in solid tumors. Tumor-associated macrophages (TAMs) play a dominant role in cancer-related inflammation and serve as crucial regulators in tumorigenesis. In GC, especially in diffuse GC, a high abundance of M2 macrophages may contribute to an immunosuppressive phenotype [43, 44]. Currently, there are no effective therapeutic targets and predictive indicators for TAMs. Therefore, we aim to identify key genes associated with TAM and discover macrophage-related targets for treatment and clinical prediction. We firstly screened for macrophage markers associated with GC prognosis. Subsequently, we identified gene modules related to these markers and established a prognostic model using these module genes with Weighted Correlation Network Analysis (WGCNA). Through LASSO analysis, we identified 13 macrophage-associated genes that are significantly correlated with GC clinical outcomes, including CPNE8, AKR1B1, MARCKS, ANXA5, SERPINE1, GAMT, SNCG, MATN3, SLC7A2, CYTL1, LIN7A, GJA1, APOD.

In the theory of immune surveillance, immune cells are believed to have the ability to recognize and eliminate tumor cells [45]. However, tumor can also evade immune system attacks through a series of immune escape mechanisms [46, 47]. The high-risk group composed of these 13 genes was significantly associated with macrophage immune infiltration in GC, as revealed by various immune infiltration analyses. This suggests that these 13 genes might impact the polarization of macrophages and influence the prognosis of GC patients. In existing research, macrophages expressing AKR1B1 promoted GC progression by regulating the microenvironment [48]; MARCKS was involved in macrophage infiltration and polarization through Ca2^+^ and PIP3 signaling [49, 50]; low expression of SLC7A2 was correlated with reduced immune infiltration [51];CYTL1 acted as a potential cell factor capable of recruiting macrophages [52]; GJA1 could serve as an M2 macrophage-associated prognostic gene in GC [53]; APOD was found to be a component that could predict overall survival and M2 macrophage infiltration in GC [54]; ANXA5 promoted the transition of macrophages from M1 to M2 through PKM2 [18]. These research findings confirm our analysis results, showing a significant correlation between the selected genes and TAM infiltration and polarization.

Gene mutations and tumor mutation burden play a crucial role in predicting immune therapy responses [55, 56]. In our study, we analyzed the relationship between the gene set of 13 prognostic genes and gene mutations. Additionally, we investigated the somatic SNV and mutation frequencies of these 13 genes. The analysis results are consistent with subsequent immune therapy response, where the high-risk group exhibited lower responsiveness to immune therapy. AKR1B1, functioning as a TNF-α downstream gene, could stratify GC patients and predict immune therapy responses [57]. ANXA5, serving as a marker for macrophage subsets, could predict immune therapy responses in endometrial cancer [58]. GAMT served as one of the components to predict the sensitivity of colorectal adenocarcinoma patients to immune therapy targeting PD-1 and CTLA-4 [59]. The downregulation of SLC7A2 upregulated CXCL1 through the PI3K/Akt/NF-κB pathway, recruiting bone marrow-derived suppressor cells and exerted a tumor immune inhibitory effect [60]. Upregulation of CYTL1 was associated with M2 macrophage infiltration and immune exhaustion in GC [61]. GJA1 was considered to potentially play a significant role in melanoma and could predict patients’ response to PD-1 immunotherapy [62]. APOD was used to construct a risk model for predicting immune infiltration and immunotherapy in cervical cancer [63]. In various studies, distinct immunotherapy drugs have demonstrated varying efficacy among GC patients at different stages. This diversity makes predicting the clinical effectiveness of ICIs challenging. Consequently, it has become essential to explore potential markers that can help assess patients who are likely to respond positively to ICIs therapy.

In various analyses encompassing disease progression, drug resistance development, and immunotherapeutic response prediction, the ANXA5 molecule stands out due to its prominent role. In light of this, we postulated that ANXA5 holds the potential to emerge as a pivotal predictive indicator. To substantiate this hypothesis, we conducted experiments involving the downregulation of ANXA5 expression, revealing a substantial impact on the process of angiogenesis. Notably, this observation aligns seamlessly with the pathway enrichment analysis findings within the high-risk group. The aforementioned research outcomes robustly endorse the latent predictive value of ANXA5. The impact of ANXA5 on angiogenesis may be mediated through the facilitation of AKT and ERK phosphorylation pathways [64]. This mechanistic insight could shed light on novel therapeutic avenues for modulating angiogenesis-related processes.

The occurrence of tumors is intricately linked to the generation of new blood vessels [65]. Antibodies and inhibitors targeting VEGF and VEGFR have undergone testing in GC. Contrary to standalone chemotherapy, the combination of Bevacizumab (anti-VEGF) and chemotherapy did not confer a survival advantage for advanced GC patients [66]. In contrast, Ramucirumab (a selective VEGFR2 monoclonal antibody) demonstrated the ability to improve clinical outcomes in advanced GC [67]. The impact of Ramucirumab on TAM has been observed, with its inhibition of VEGFR2 potentially leading to reduced TAM immune infiltration, as well as decreased release of cytokines and chemokines, thereby restraining tumor growth and proliferation [68]. The specific role of ANXA5 in the process of treating advanced GC with Ramucirumab remains unclear and warrants further investigation for confirmation.

Phosphatidylserine (PS) is located in the inner layer of the cytoplasmic membrane of healthy cells. During apoptosis, PS is translocated to the outer layer of the cell membrane, facilitating the clearance of apoptotic cells by phagocytes. Despite elevated levels of PS in tumor cells, they do not undergo apoptosis. ANXA5 binds to PS with high affinity, mediating the clearance of PS + apoptotic cells by immune cells [69]. Exploiting these features, Kang et al. designed ANXA5-peptide fusion to enhance the immunogenicity of tumor cells, thereby strengthening the ability of immune cells to recognize tumor cells [70]. Previous studies have also shown that ANXA5, in a synergistic manner, promotes the TCR signaling of homologous peptide-major histocompatibility complex (pMHC), leading to enhanced activation of lymphocytes [71]. Therefore, ANXA5 may serve as a potential target for immunotherapy as an immune checkpoint inhibitor responsive to treatment.

Our study has the potential to provide novel insights into unraveling mechanisms of action of macrophage-related genes in GC and to furnish substantive groundwork for the construction of predictive models of GC. This achievement could feasibly exert a significant impact on prognostic markers and immune resistance gene predictions, offering fresh avenues for the formulation of personalized therapeutic strategies. Finally, it is worth noting that ANXA5 may potentially impact the prognosis of GC patients and immune therapy response through its influence on the angiogenesis phenotype.

### Electronic supplementary material

Below is the link to the electronic supplementary material.

Supplementary Material 1

**Supplementary Figure 1**. Single-factor regression analysis to screen for immune cell marker genes that are associated with the prognosis of gastric cancer. **Supplementary Figure 2**. Identification of key modules associated with macrophages. (A)Visual representation of hierarchical clustering analysis results, including a dendrogram (above) for gene hierarchical clustering and gene modules (below). (B) Gene module correlation heatmap. (C) Macrophage infiltration scoring heatmap. **Supplementary Figure 3**. Constructing prognosis model using LASSO regression. (A)Prognostic related genes and their coefficients related to overall survival (OS). (B) Distribution of risk scores, survival times, and gene expression information in the prognostic model in the TCGA training set. (C) Regression coefficients of various variables in the multifactorial Cox regression incorporating clinical variables and risk scores. (D-E) ROC and KM survival curve analysis based on multifactorial risk scores. (F-G) Univariate and multivariate Cox regression analyses assessing the predictive value of risk scores for gastric cancer prognosis. **Supplementary Figure 4**. Evaluating the prognostic model constructed with macrophage-related genes in the validation dataset.(A) A nomogram based on the Cox model was verified to predict 1-5 year overall survival (OS); (B)The calibration curve of the overall predicted 1-5 year OS nomogram in validation dataset; (C)The coefficients of each variable were obtained according to the result of the multivariate Cox model that integrated clinical variables and 13-gene risk score; (D-E) Univariate and multivariate Cox regression analyses found that the risk score was an independent prognostic factor when clinical variables were included; (F-G) Kaplan-Meier survival analysis (G) and time-dependent ROC analysis (F) were performed based on median risk scores of each sample; (H-J) Decision curve analysis (DCA) for 1, 3, and 5-year was conducted to verified the application value of the model in different prediction periods. **Supplementary Figure 5**. Immune cell infiltration analysis in different risk groups. (A-L) Evaluation of immune infiltration characteristics in high- and low-risk groups using 12 different analysis algorithms. *p < 0.05; **p < 0.01; ***p < 0.001. **Supplementary Figure 6**. Different risk group mutation maps. (A) Waterfall plot of gene mutations in different groups. (B-C) Analysis of SNVs and mutation frequencies of prognostically relevant genes. (D-F) Analysis of tumor purity, homologous recombination deficiency (HRD), and loss of heterozygosity (LOH) in different risk groups. **Supplementary Figure 7**. Prognostic-related genes and their impact on prognosis, along with expression analysis. (A-M) Analysis of the prognostic impact of 13 genes on gastric cancer patient outcomes. (N) Visualization of Hazard Ratio (HR) outcomes from the survival analysis using a bar chart with error bars. (O) Expression analysis of the 13 prognostic-related genes in the gastric cancer datasets from TCGA and TCGA-GTEx. **Supplementary Figure 8**. Original images of gels.

## Data Availability

The datasets analysed during the current study are available in the TCGA and GEO. [TCGA-STAD, GSE62254_ACRG, GSE13861, GSE15459, GSE26901, GSE26899, GSE34942, GSE84433, GSE62254, GSE84437, GSE28541, GSE84426, GSE29272, GSE26253, GSE14208, GSE57303, GSE183136, GSE38749, Riaz cohort 2018(GSE91061), Ascierto cohort 2016(GSE79691), Lauss cohort 2017(GSE100797), Gao cohort 2018(GSE115821), Cho cohort 2020 (GSE126044)]
