# Supplementary figures and images for: The macrophage-associated prognostic gene ANXA5 promotes immunotherapy resistance in gastric cancer through angiogenesis
Source: BMC Cancer. 2024 Jan 29;24:141. doi: 10.1186/s12885-024-11878-7 (PMC10823665; doi:10.1186/s12885-024-11878-7)

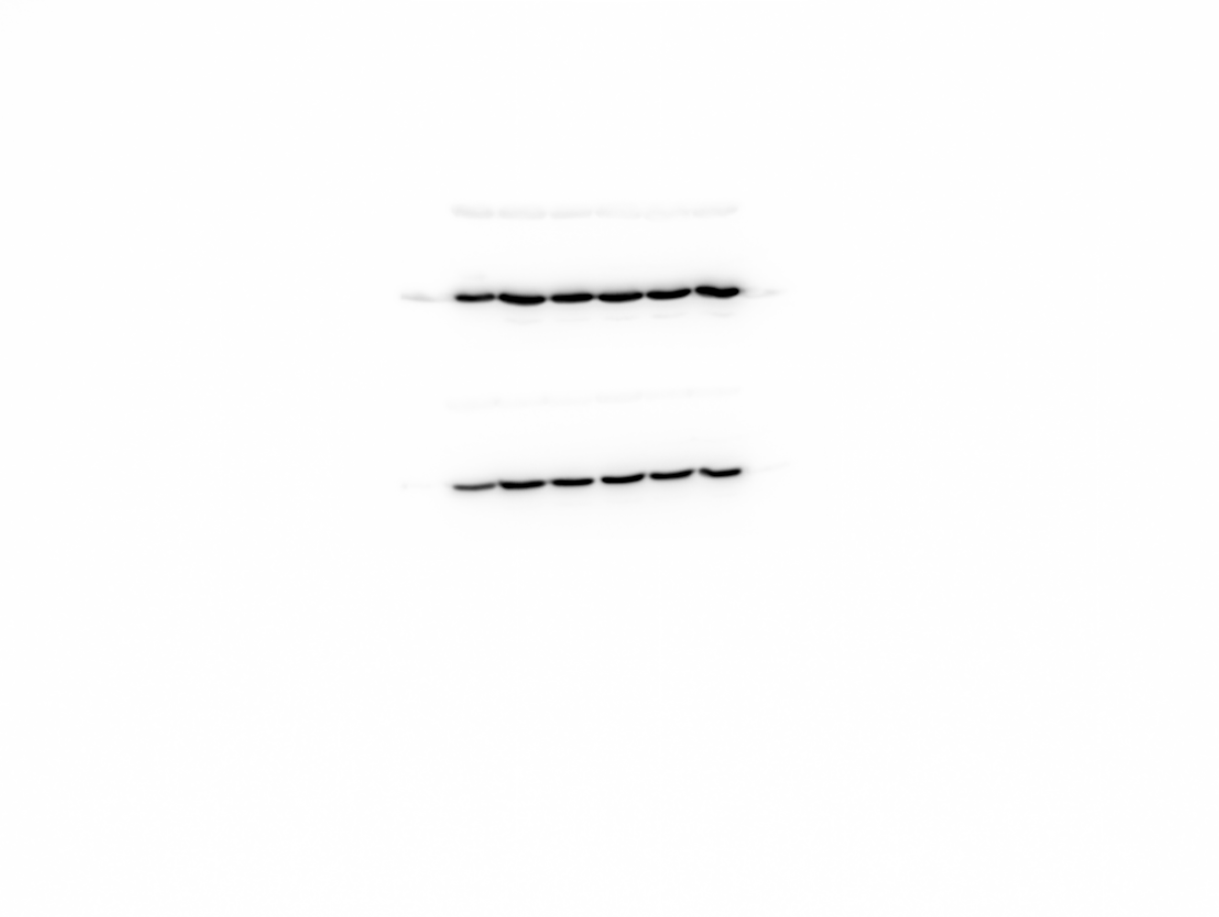

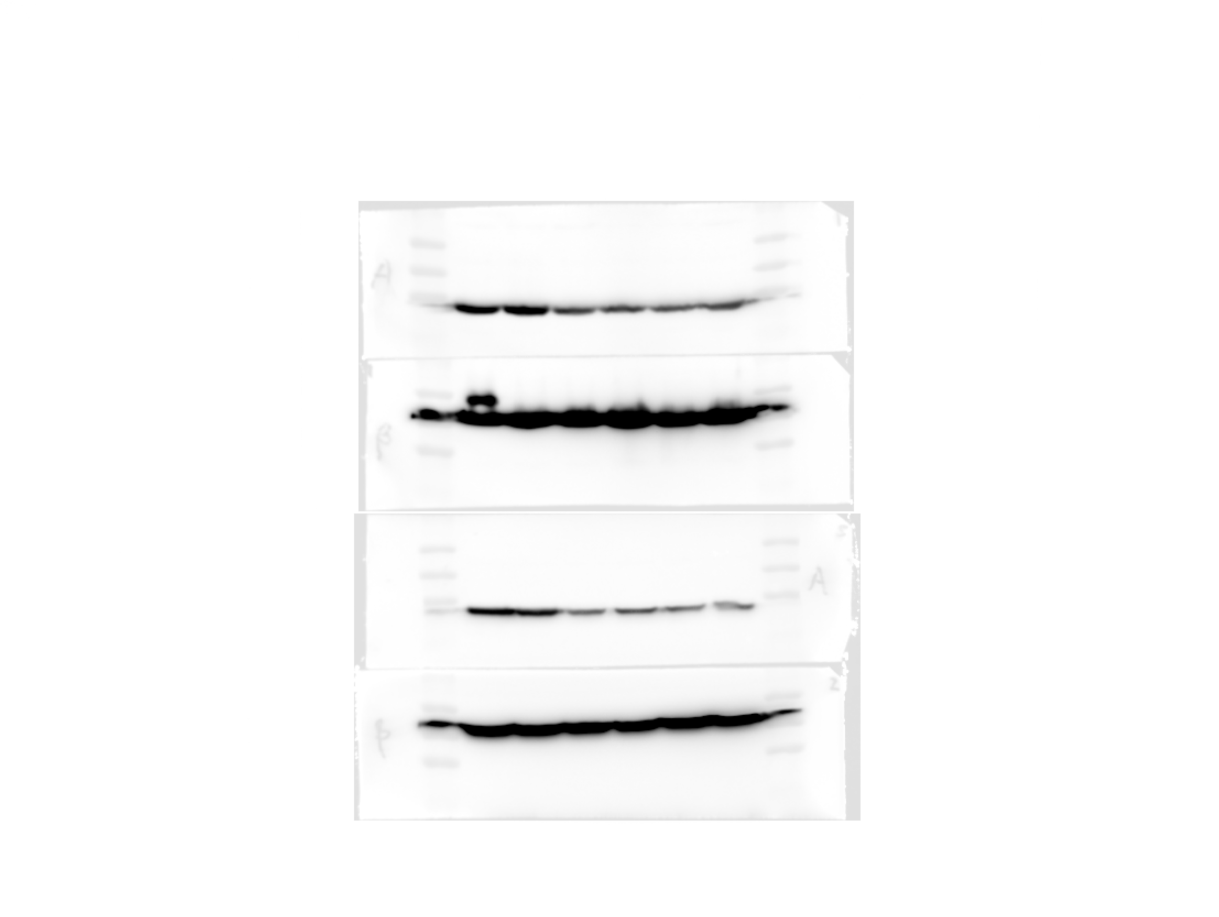

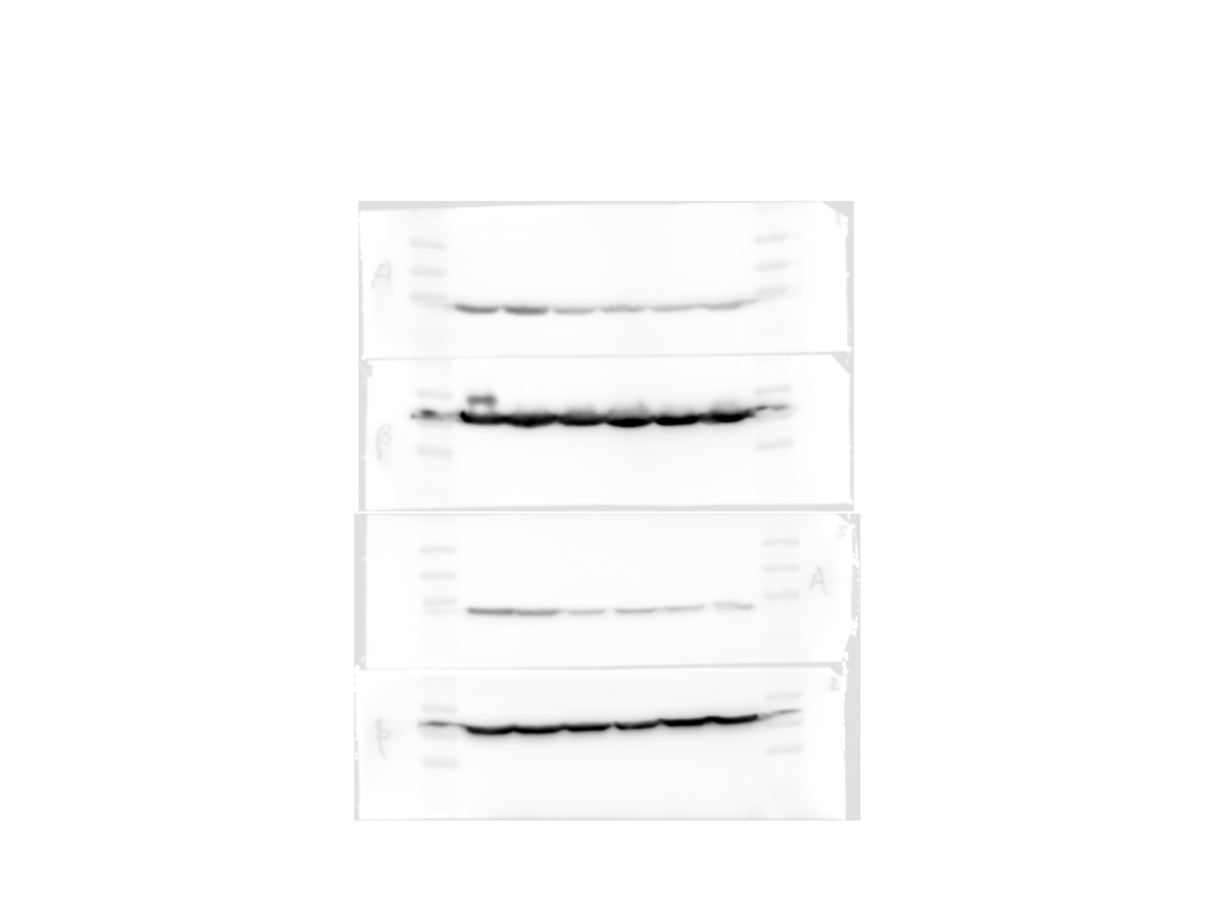

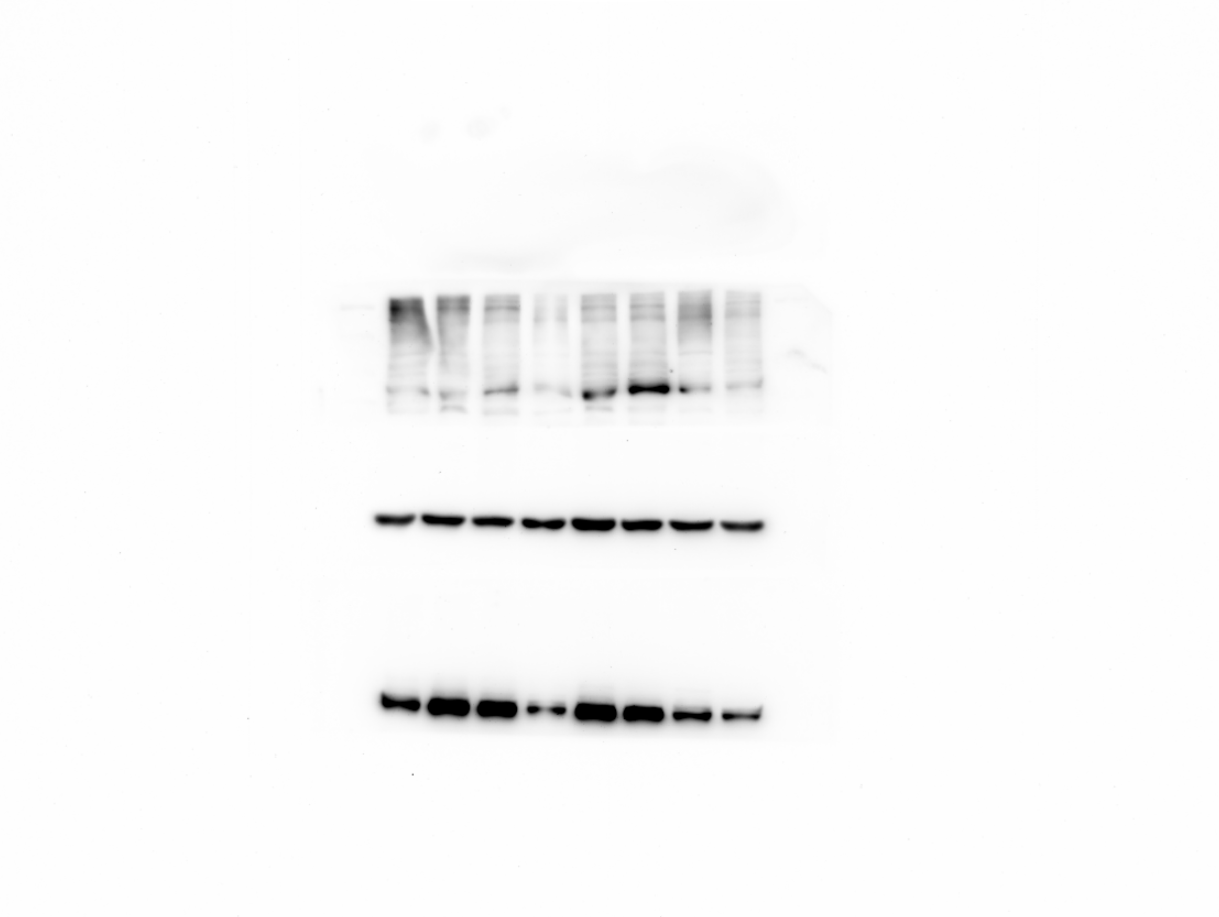

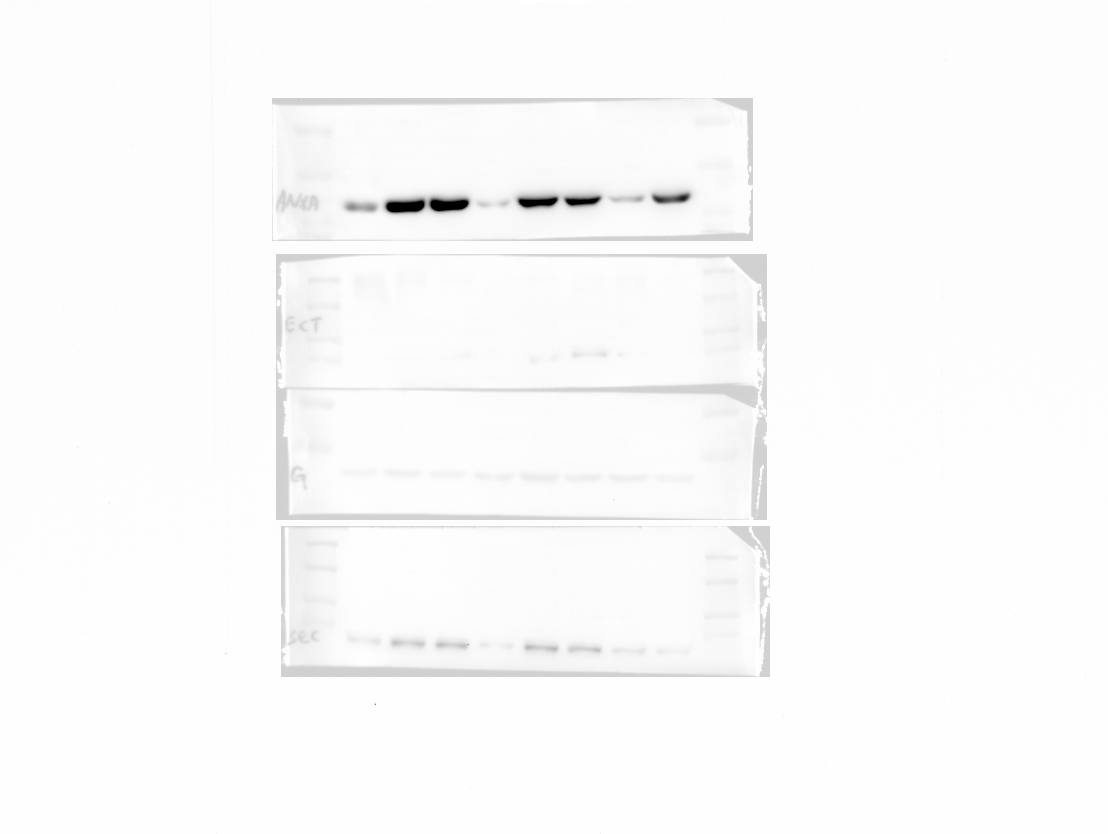

Supplement: Supplementary file 1 — Supplementary Material 1 [file 12885_2024_11878_MOESM1_ESM.docx]
