# Supplementary material for: The macrophage-associated prognostic gene ANXA5 promotes immunotherapy resistance in gastric cancer through angiogenesis
Source: BMC Cancer. 2024 Jan 29;24:141. doi: 10.1186/s12885-024-11878-7 (PMC10823665; doi:10.1186/s12885-024-11878-7)
Supplement: Supplementary file 2 — Supplementary Figure 1. Single-factor regression analysis to screen for immune cell marker genes that are associated with the prognosis of gastric cancer. Supplementary Figure 2. Identification of key modules associated with macrophages. (A)Visual representation of hierarchical clustering analysis results, including a dendrogram (above) for gene hierarchical clustering and gene modules (below). (B) Gene module correlation heatmap. (C) Macrophage infiltration scoring heatmap. Supplementary Figure 3. Constructing prognosis model using LASSO regression. (A)Prognostic related genes and their coefficients related to overall survival (OS). (B) Distribution of risk scores, survival times, and gene expression information in the prognostic model in the TCGA training set. (C) Regression coefficients of various variables in the multifactorial Cox regression incorporating clinical variables and risk scores. (D-E) ROC and KM survival curve analysis based on multifactorial risk scores. (F-G) Univariate and multivariate Cox regression analyses assessing the predictive value of risk scores for gastric cancer prognosis. Supplementary Figure 4. Evaluating the prognostic model constructed with macrophage-related genes in the validation dataset.(A) A nomogram based on the Cox model was verified to predict 1-5 year overall survival (OS); (B)The calibration curve of the overall predicted 1-5 year OS nomogram in validation dataset; (C)The coefficients of each variable were obtained according to the result of the multivariate Cox model that integrated clinical variables and 13-gene risk score; (D-E) Univariate and multivariate Cox regression analyses found that the risk score was an independent prognostic factor when clinical variables were included; (F-G) Kaplan-Meier survival analysis (G) and time-dependent ROC analysis (F) were performed based on median risk scores of each sample; (H-J) Decision curve analysis (DCA) for 1, 3, and 5-year was conducted to verified the application value of [file 12885_2024_11878_MOESM2_ESM.docx]

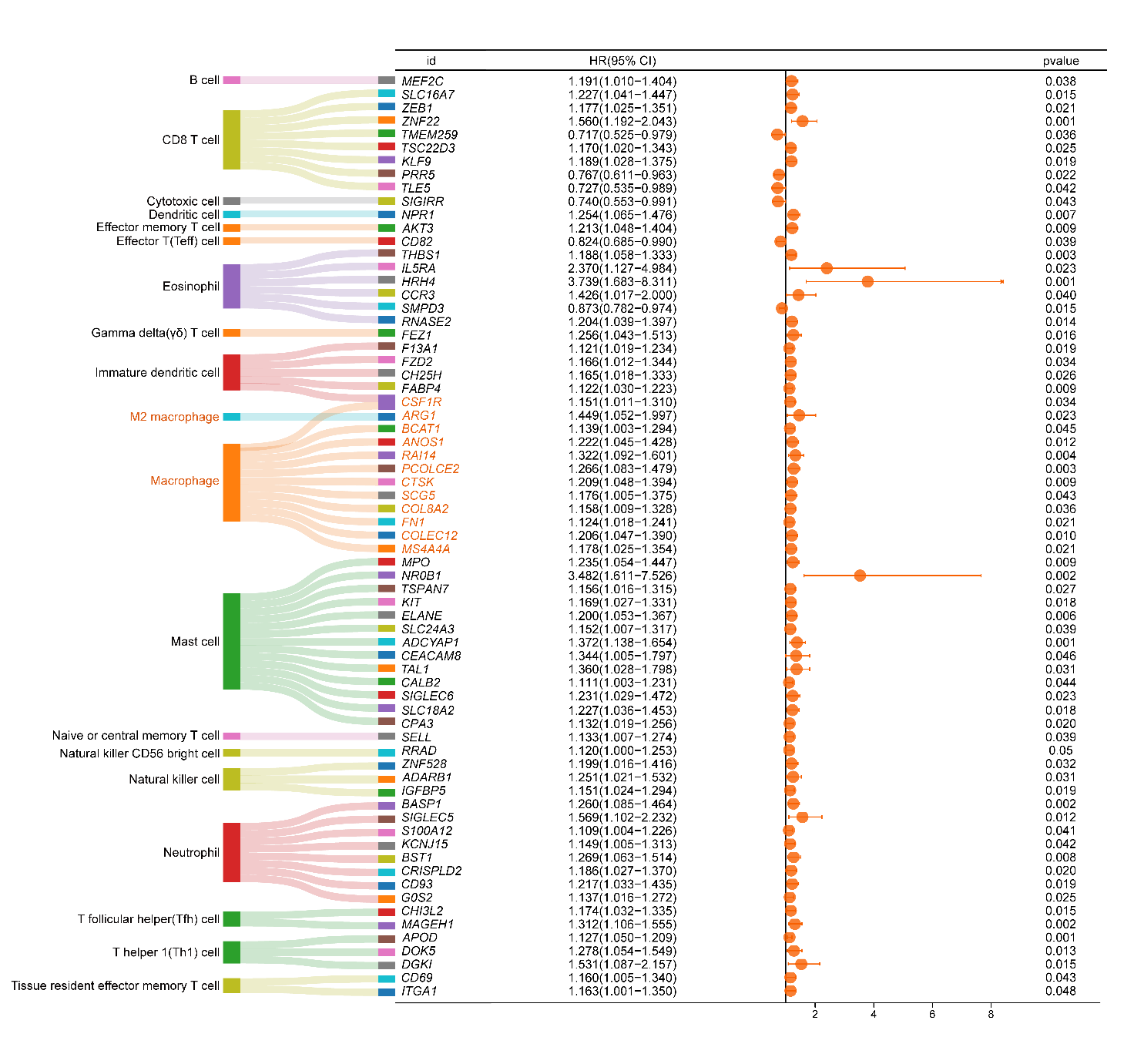


**Supplementary Figure 1. Single-factor regression analysis to screen for immune cell marker genes that are associated with the prognosis of gastric cancer.**


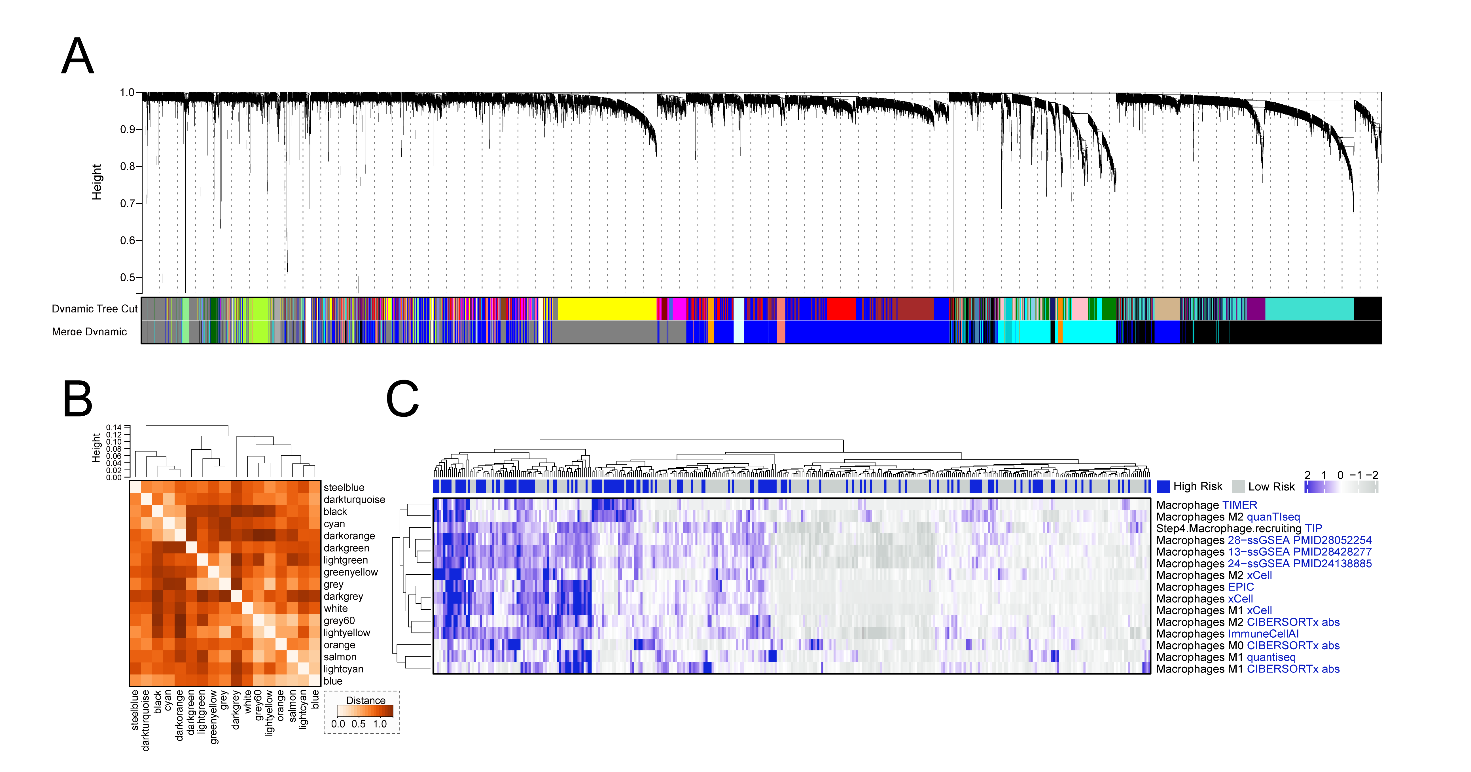


**Supplementary Figure 2. Identification of key modules associated with macrophages.** (A)Visual representation of hierarchical clustering analysis results, including a dendrogram (above) for gene hierarchical clustering and gene modules (below). (B) Gene module correlation heatmap. (C) Macrophage infiltration scoring heatmap.


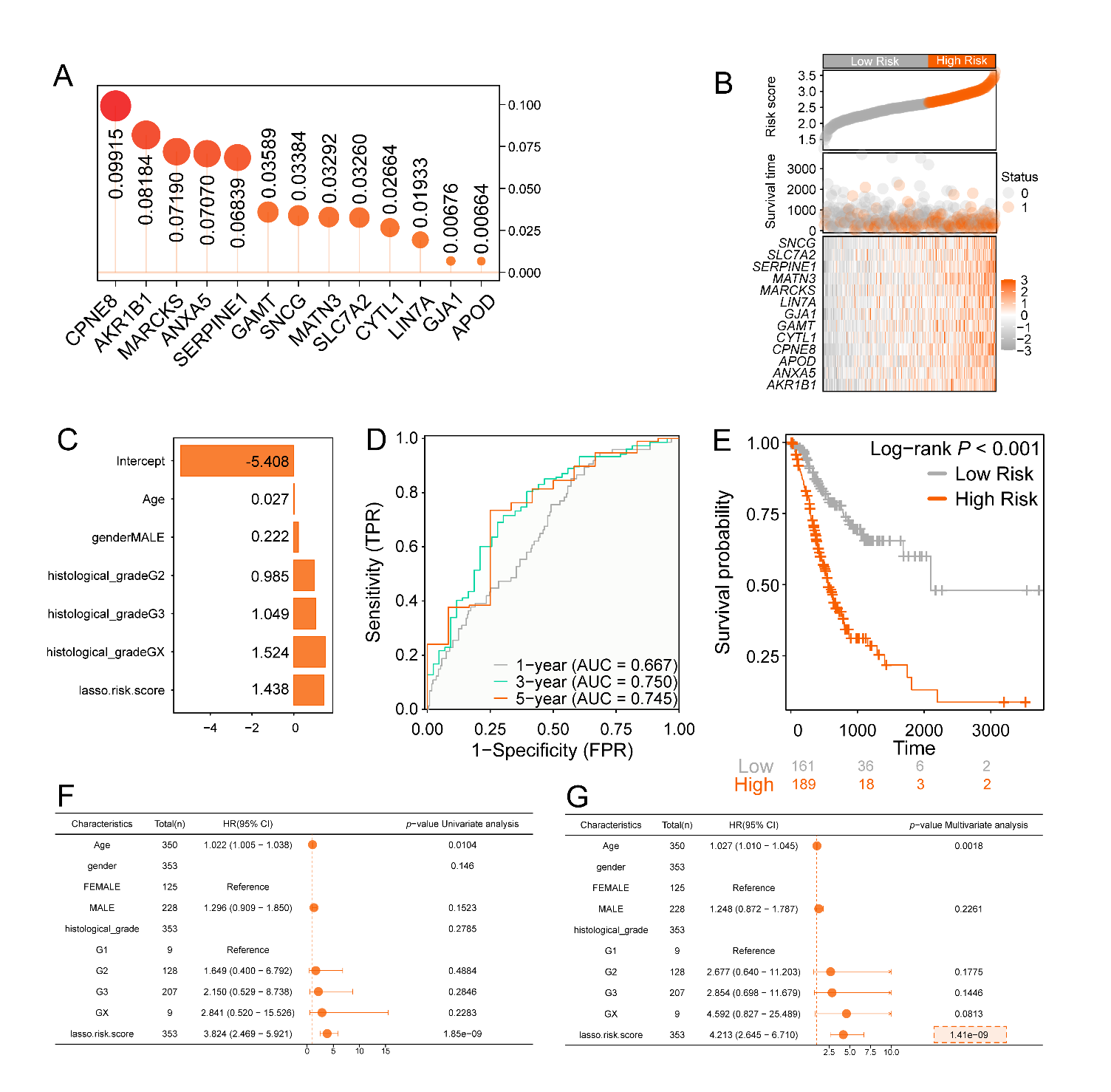


**Supplementary Figure 3. Constructing prognosis model using LASSO regression.** (A)Prognostic related genes and their coefficients related to overall survival (OS). (B) Distribution of risk scores, survival times, and gene expression information in the prognostic model in the TCGA training set. (C) Regression coefficients of various variables in the multifactorial Cox regression incorporating clinical variables and risk scores. (D-E) ROC and KM survival curve analysis based on multifactorial risk scores. (F-G) Univariate and multivariate Cox regression analyses assessing the predictive value of risk scores for gastric cancer prognosis.


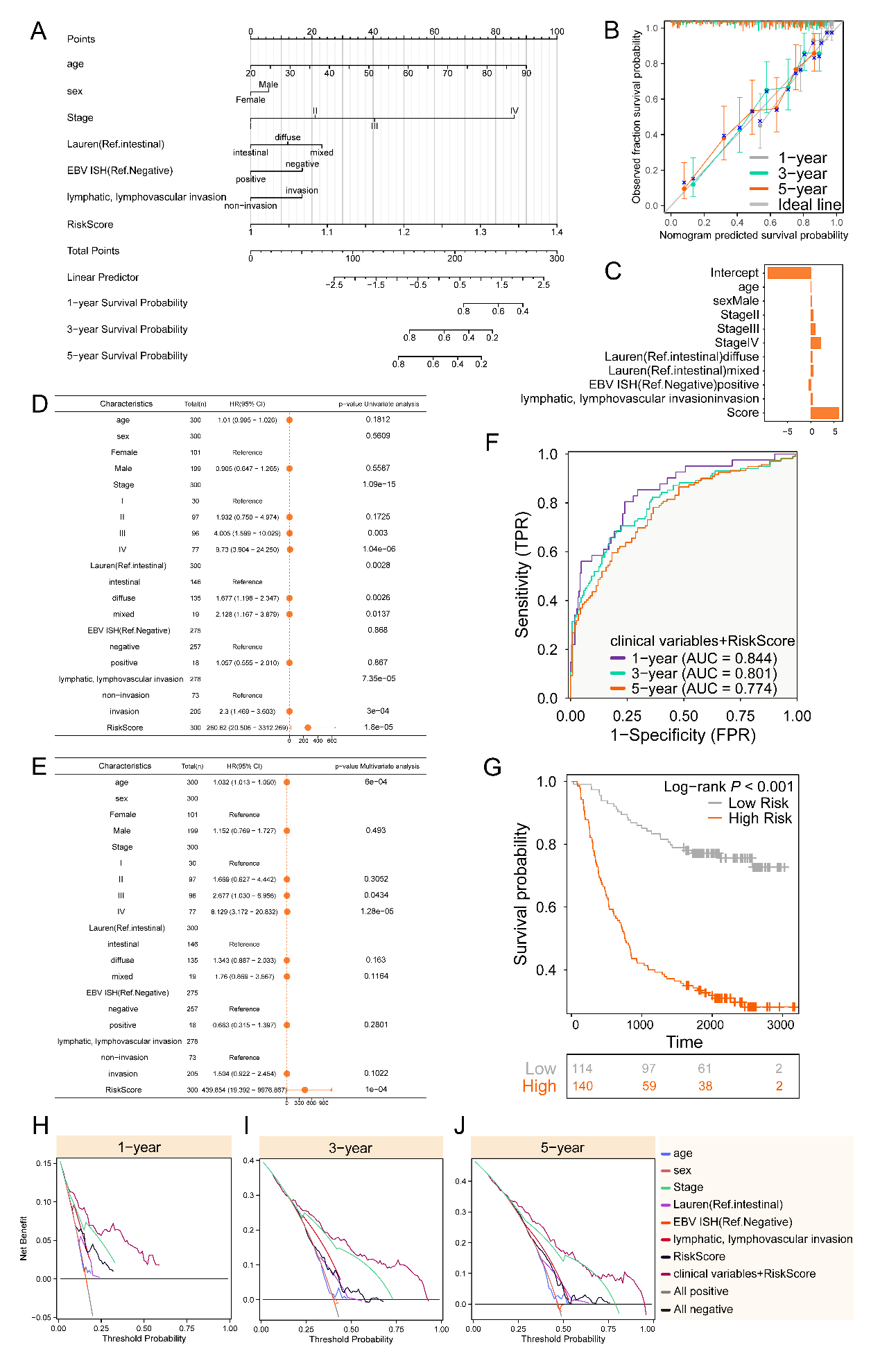


**Supplementary Figure 4. Evaluating the prognostic model constructed with macrophage-related genes in the validation dataset.**(A) A nomogram based on the Cox model was verified to predict 1-5 year overall survival (OS); (B)The calibration curve of the overall predicted 1-5 year OS nomogram in validation dataset; (C)The coefficients of each variable were obtained according to the result of the multivariate Cox model that integrated clinical variables and 13-gene risk score; (D-E) Univariate and multivariate Cox regression analyses found that the risk score was an independent prognostic factor when clinical variables were included; (F-G) Kaplan-Meier survival analysis (G) and time-dependent ROC analysis (F) were performed based on median risk scores of each sample; (H-J) Decision curve analysis (DCA) for 1, 3, and 5-year was conducted to verified the application value of the model in different prediction periods.
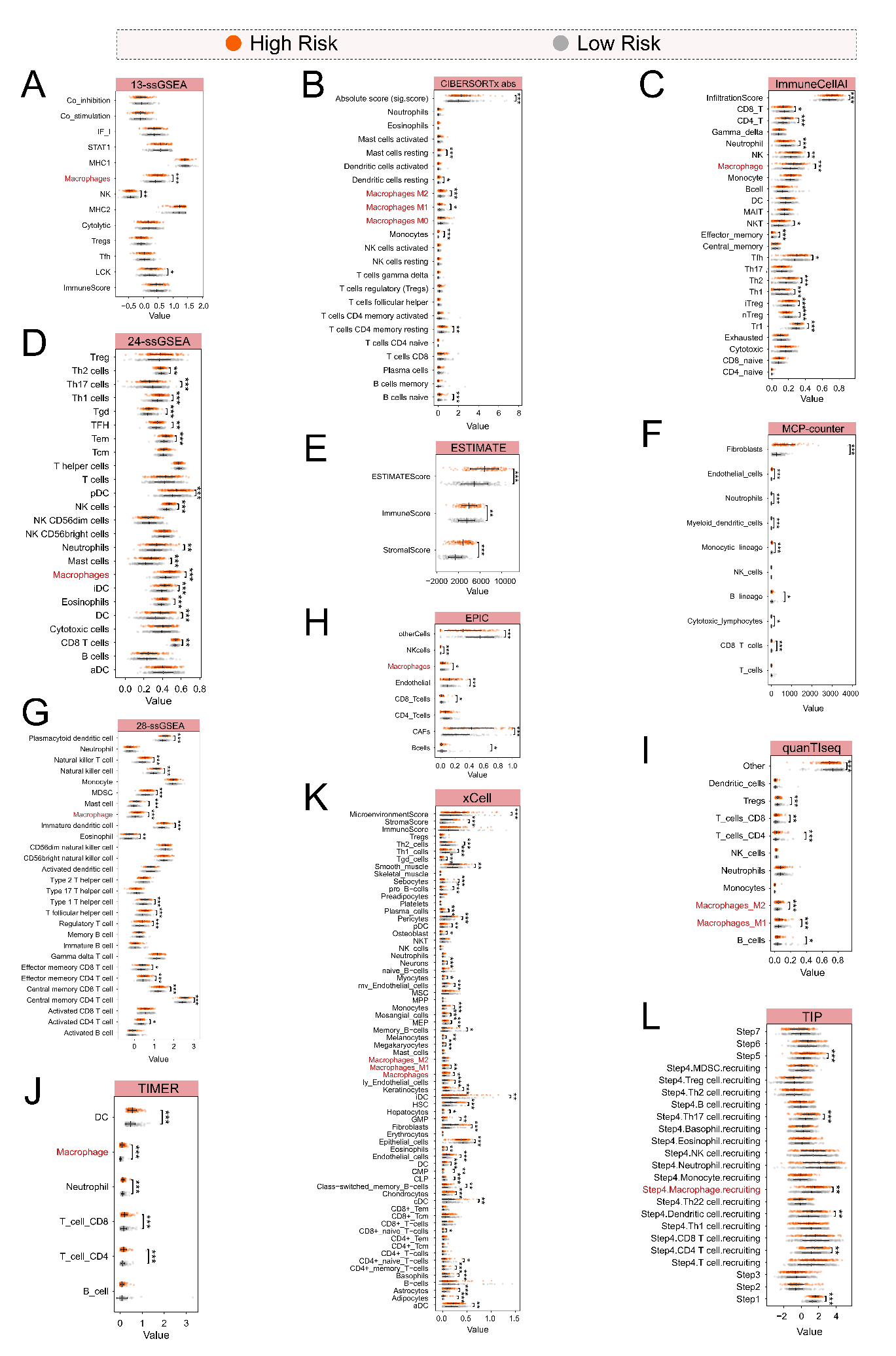


**Supplementary Figure 5. Immune cell infiltration analysis in different risk groups.** (A-L) Evaluation of immune infiltration characteristics in high- and low-risk groups using 12 different analysis algorithms. *p< 0.05; **p< 0.01; ***p< 0.001.


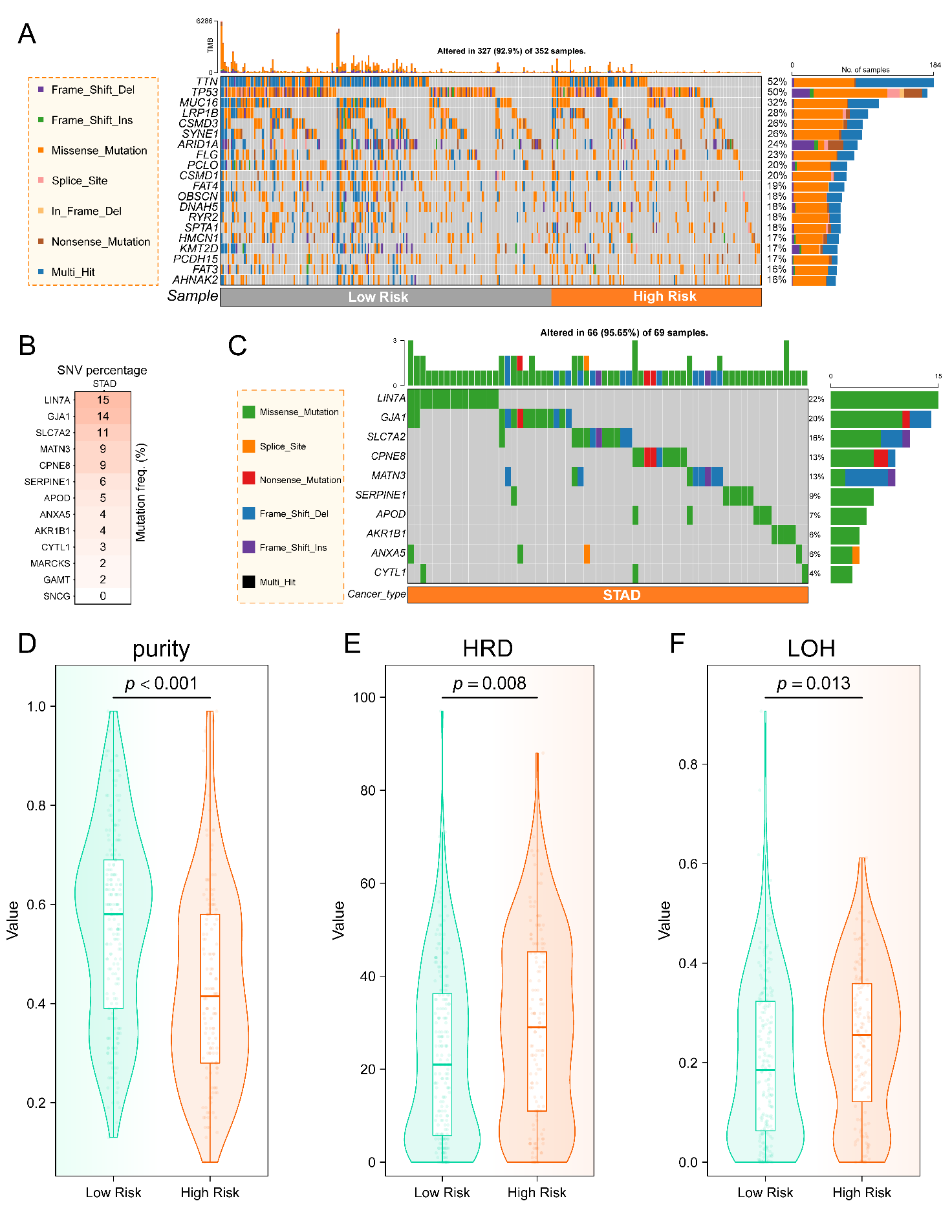


**Supplementary Figure 6. Different risk group mutation maps.** (A) Waterfall plot of gene mutations in different groups. (B-C) Analysis of SNVs and mutation frequencies of prognostically relevant genes. (D-F) Analysis of tumor purity, homologous recombination deficiency (HRD), and loss of heterozygosity (LOH) in different risk groups


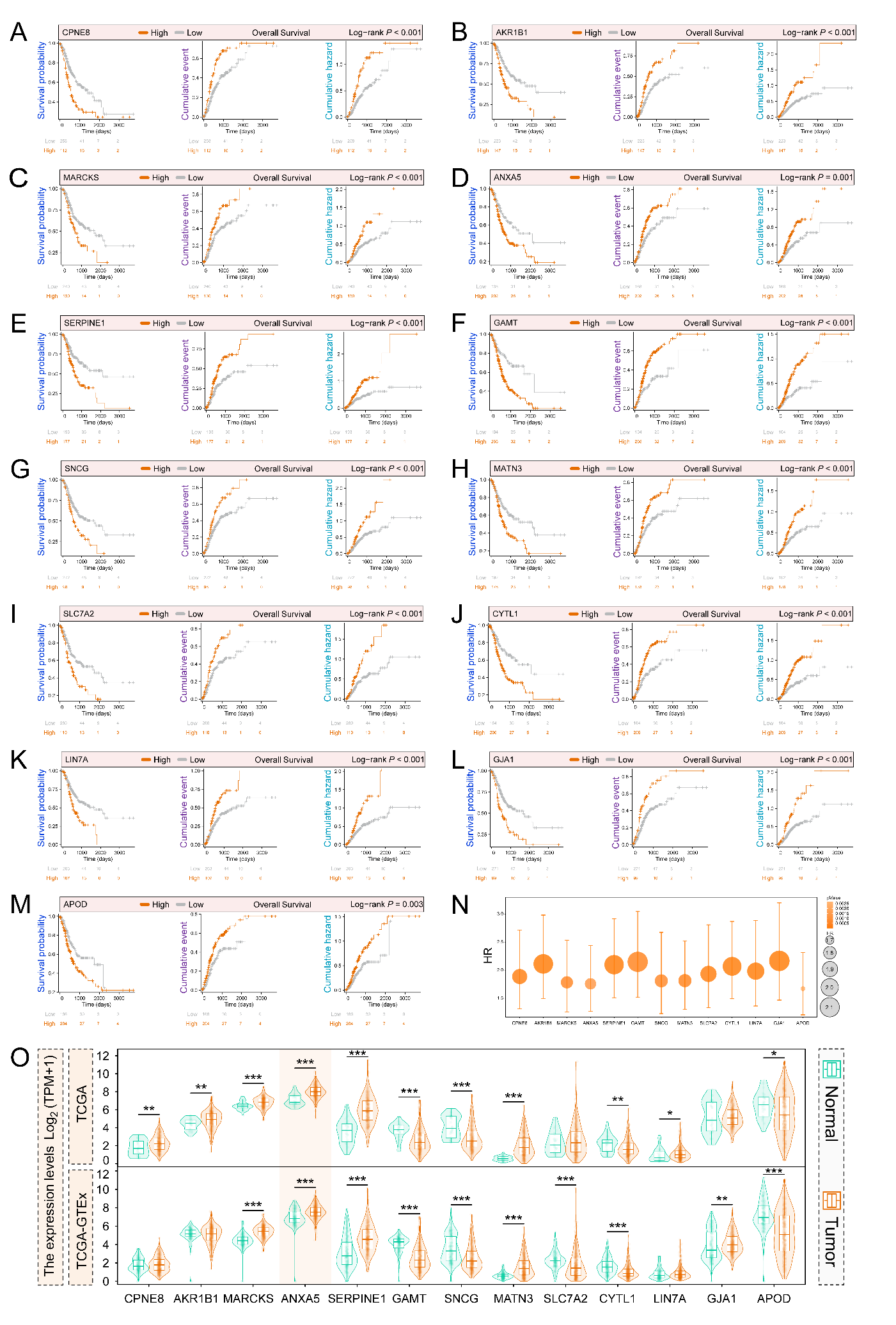


**Supplementary Figure 7. Prognostic-related genes and their impact on prognosis, along with expression analysis.** (A-M) Analysis of the prognostic impact of 13 genes on gastric cancer patient outcomes. (N) Visualization of Hazard Ratio (HR) outcomes from the survival analysis using a bar chart with error bars. (O) Expression analysis of the 13 prognostic-related genes in the gastric cancer datasets from TCGA and TCGA-GTEx.


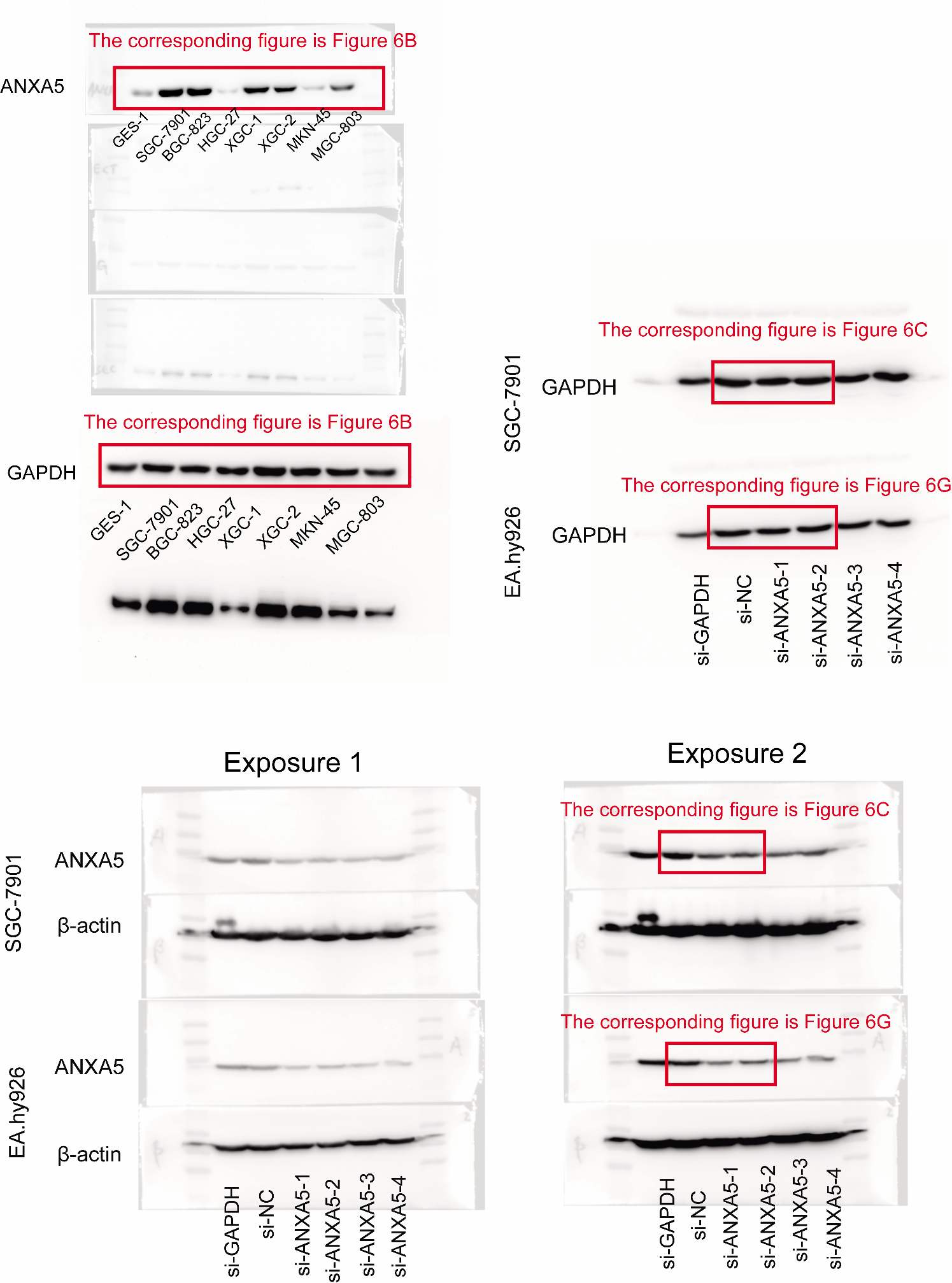
 **Supplementary Figure 8.** **Original images of gels.**
